# The slow de‐implementation of non‐evidence‐based treatments in low back pain hospital care—Trends in treatments using Dutch hospital register data from 1991 to 2018

**DOI:** 10.1002/ejp.2052

**Published:** 2022-11-12

**Authors:** Pieter Coenen, Astrid de Wind, Peter van de Ven, Marianne de Maaker‐Berkhof, Bart Koes, Rachelle Buchbinder, Jan Hartvigsen, Johannes (Han) R. Anema

**Affiliations:** ^1^ Department of Public and Occupational Health, Amsterdam UMC Amsterdam Public Health Research Institute, Vrije Universiteit Amsterdam Amsterdam the Netherlands; ^2^ Department of Public and Occupational Health, Amsterdam UMC Coronel Institute of Occupational Health, Amsterdam Public Health Research Institute, University of Amsterdam Amsterdam the Netherlands; ^3^ Department of Epidemiology and Data Science, Amsterdam UMC Vrije Universiteit Amsterdam the Netherlands; ^4^ Department of Sports Science and Clinical Biomechanics University of Southern Denmark Odense Denmark; ^5^ Department of General Practice, Erasmus MC University Medical Center Rotterdam Rotterdam the Netherlands; ^6^ Department of Epidemiology and Preventive Medicine School of Public Health and Preventive Medicine, Monash University Melbourne Australia; ^7^ Monash Department of Clinical Epidemiology Cabrini Health Malvern Victoria Australia; ^8^ Chiropractic Knowledge Hub Odense Denmark

## Abstract

**Background:**

Low back pain (LBP) is the leading cause of disability worldwide and has an excessive societal burden. Accumulating evidence has shown that some medical approaches such as imaging in absence of clear indications, medication and some invasive treatments may contribute to the problem rather than alleviating it.

**Objectives:**

To determine the extent of de‐implementation of non‐evidence‐based hospital treatments for LBP care in the Netherlands in the last three decades.

**Methods:**

Using a register‐based population‐level observational study with Dutch hospital data, providing a nearly complete coverage of hospital admissions in the Netherlands in 1991–2018, we assessed five frequently applied non‐evidence‐based hospital treatments for LBP. Time trends in treatment use (absolute and per 100,000 inhabitants) were plotted and analysed using Poisson regression.

**Results:**

The use of bed rest for non‐specific LBP and hernia nuclei pulposi, and discectomy for spinal stenosis decreased 91%, 81% and 86% since the availability of evidence/guidelines, respectively. De‐implementation, beyond 84%, was reached after 18 and 17 years for bed rest for non‐specific LBP and discectomy respectively, while it was not reached after 28 years for bed rest for hernia nuclei pulposi. For spinal fusion and invasive pain treatment, there was an initial increase followed by a reduction. Overall, these treatments reduced by 85% and 75%, respectively.

**Conclusions:**

In the Netherlands, de‐implementation of five non‐recommended hospital LBP treatments, if at all, took several decades. Although de‐implementation was substantial, slow de‐implementation has likely resulted in considerable waste of resources and avoidable harm to many patients in Dutch hospitals.

**Significance:**

Medically intensive approaches to low‐back pain care contribute to the high societal burden of this disease. There have been calls to avoid such care. Using Dutch hospital data, we showed that de‐implementation of five non‐recommended hospital low‐back pain treatments, if at all, took several decades (i.e. ≥17 years) after availability of evidence and guidelines. Slow de‐implementation has likely resulted in considerable waste of resources and avoidable harm to hospital patients; better ways for de‐implementation of non‐evidence‐based care are needed.

## 1 INTRODUCTION

Low back pain (LBP) affects most people at some point in their life and is the leading cause of disability worldwide (GBD 2019 Diseases and Injuries Collaborators, 2020). Consequently, the societal economic burden of LBP is enormous (Dagenais et al., 2008). In the US, medical costs for LBP are the highest of all diseases, estimated US$134.5billion in 2016. In the Netherlands, the most recent numbers stem from 2007, when annual total LBP costs were estimated €3.5billion (Lambeek et al., 2011).

One reason for the high economic burden of LBP is the medically intensive approach to care, with prevalent unwarranted imaging, pain‐medication and invasive treatments (Hartvigsen et al., 2018). Systematic reviews have indicated either a paucity of evidence or evidence for marginal, no beneficial or even harmful effects of medical approaches such as bed rest (Dahm et al., 2010), imaging in the absence of a clear indication (van der Windt et al., 2010), surgery (e.g. spinal stenosis (Machado et al., 2016) and fusion (Harris et al., 2018)) and pain medication (e.g. opioids (Chaparro et al., 2014), non‐steroidal non‐inflammatory drugs (Enthoven et al., 2016) and anti‐depressants (Ferreira et al., 2021)). Conversely, evidence suggests most cases of LBP do not require active treatment or can be managed by non‐medicalizing approaches (Foster et al., 2018). This is mainly the case for 95% of LBP patients for which no clear patho‐anatomical cause of their pain can be found, whose pain is labelled non‐specific (Maher et al., 2017).

International experts highlighted that one of the key challenges in managing LBP is to avoid ‘harmful or useless treatments’, while ensuring high‐quality care for those who need it (Buchbinder et al., 2018). Indeed, (inter)national guidelines now emphasize that first line care should focus less on pharmacological treatments while many diagnostic, medical and invasive treatments are no longer recommended for routine care (Foster et al., 2018). Despite mounting evidence and available guidelines, it appears difficult to de‐implement non‐recommended treatments.

‘Innovators’ and ‘early adapters’ of new evidence together make up ~16% of clinicians, whereas for two‐thirds, adoption of evidence into practice is estimated to occur after a substantial time‐lag (Rogers, 2003) of up to 17 years (Balas & Boren, 2000). This lag reflects the time interval necessary for effective knowledge dissemination and translation, and corresponding uptake and scaling into policy and practice (Penfield et al., 2014). The so‐called ‘laggards’ are the last 16% of clinicians to adopt new evidence (Rogers, 2003). There are many barriers and facilitators for de‐implementation of non‐evidence‐based treatment (Hall et al., 2019). These can be classified into social (e.g. wanting to maintain a good relationship with patients by addressing their preferences [Schers et al., 2000]), beliefs and (lack of) knowledge (Schers et al., 2001), and environmental factors (e.g. financial incentives (Traeger et al., 2019), lack of time (Slade et al., 2015) or access to treatment options (Breen et al., 2007)).

While there have been persistent calls to avoid potential harmful and/or wasteful LBP care, it is unclear whether de‐implementation has occurred due to emerging evidence and guidelines. In this study, we determined the extent of de‐implementation of five non‐evidence‐based hospital LBP treatments. To the best of our knowledge, this study is the first to investigate de‐implementation (instead of implementation) of medical procedures for low‐back pain, taking a historical perspective using national hospital register data.

## 2 METHODS

### 2.1 Study design

We described results from a register‐based population‐level observational study using Dutch hospital data from 1991 to 2018. We conducted and reported this study in accordance with the *Strengthening the Reporting of Observational Studies in Epidemiology* (STROBE) guidelines (von Elm et al., 2007).

### 2.2 Identification of non‐evidence‐based treatments

In the time‐period 1991–2018, several international publications synthesizing new evidence regarding LBP management were published (e.g., Cochrane reviews [Gibson, Grant, et al., 2000; Gibson, Waddell, et al., 2000; Hilde et al., 2002; Nelemans et al., 1999]), which often formed the basis of guideline developed. We conducted a systematic search in databases for mono−/multi‐disciplinary and primary/secondary health care provider organization LBP guidelines, yielding a total of 17 Dutch national guidelines (File S1). The first guidelines on the topic were published from 1995 onwards (CBO, 1995, 2003, 2008; Faas et al., 1996). Both evidence and guidelines indicated either a paucity of evidence for some interventions, or evidence of no or marginal benefits or even harmful effects for other interventions (File S2). In some instances, the guidelines were released after the publication of the relevant Cochrane review, while for others it was the other way around. For this study, we considered treatments that were not based upon evidence as determined by the results of Cochrane reviews as non‐evidence‐based treatments, as such reviews are regarded to have a very high standard of evidence synthesis, and often form the basis of evidence underpinning clinical guidelines (Alderson & Tan, 2011). We considered the first publication containing new information regarding a treatment (either a Cochrane review or a clinical guideline) as the first landmark.

We focussed on five frequently applied hospital LBP treatments (both inpatient and outpatient) for which we considered both evidence and guidelines that emerged since 1991 supporting their de‐implementation: (1) bed rest for non‐specific LBP and (2) bed rest for hernia nuclei pulposi; (3) discectomy for spinal stenosis; (4) spinal fusion for non‐specific LBP, degenerative low back problems and spinal stenosis; and (5) invasive pain treatment for non‐specific LBP, degenerative low back problems and spinal stenosis. While the focus of this study was on low back pain, for the sake of completeness, we also included conditions that could cause upper back or neck pain.

### 2.3 Data

We used Dutch hospital register data from the Nationwide Basic Registration Hospital Care (in Dutch: *Landelijke Basisregistratie Ziekenhuiszorg*, LBZ) for the period 01 January 1991 until 31 December 2018. The LBZ registers admissions in most general and academic hospitals and some clinics, providing a nearly complete coverage of Dutch hospital admissions. All admissions are registered based on a uniform registration system, including data on admission and discharge dates and diagnosis and treatment information (as reported by the hospitals) (Dutch Hospital Data; https://www.dhd.nl). A study on the reliability of admission and discharge information showed that in 1999, 99% of this information was registered correctly (Paas & Veenhuizen, 2002). Data had 100% completeness from 2014 onwards, with incompleteness ranging from 0.3% in 2002 to 18% in 2012 (File S3), without this incompleteness being biased by medical disciplines (personal communication with LBZ).

Four categories of LBP disorders were obtained from the LBZ register: (1) hernia nuclei pulposi (herniated disc), (2) degenerative low back problems, (3) lumbar spinal stenosis, and (4) non‐specific LBP. File S4 specifies the International Classification of Diseases (ICD) version 9 and 10 codes that were used to match codes to these four categories, using the categorization developed by Cherkin and colleagues (Cherkin et al., 1992). We targeted the following treatments from the LBZ register: bed rest which we operationalized as hospital admission, invasive pain treatment (i.e. surgical treatments aimed at relieving symptoms, such as thermolysis of the spinal ganglion or dissection of the nerve root), and surgical treatments aimed at addressing the presumed pathology causing the LBP (i.e. discectomy including chemonucleolysis, spondylodesis and laminectomy). File 5 specifies which treatment codes we included together with their associated descriptions. Both diagnoses and treatments were based on categories that were relevant at the time of first data collection (i.e. in the early 1990 s) (File S2).

Yearly use (i.e. incidence) of each of the LBP categories and treatments were obtained for age groups from age 18 years onwards and then in 5‐year categories, and sex. Using demographic data from Statistics Netherlands for the population dynamics (Statistics Netherlands; https://www.opendata.cbs.nl), use was also expressed in yearly incidence per 100,000 inhabitants.

### 2.4 Analytical approach

First, we plotted time trends in absolute number of treatments from the beginning of 1991 to the end of 2018. Then, we plotted, time trends in relative treatments (per 100,000 inhabitants). We narratively described time trends in absolute and relative treatments, and expressed de‐implementation in percentage of treatments in 2018 with respect to 1991, and with respect to the year of publication of evidence/guidelines. From that percentage, and in accordance to the theory of Rogers (Rogers, 2003), we assessed the time lag for 84% of de‐implementation to take place; that is, excluding the time lag of the last 16% of clinicians (the ‘laggards’) to have adopted de‐implementation into practice.

As additional supporting analyses, we used Poisson generalized linear modelling (GLM) to compare the mean yearly use in treatments between three distinctive time‐periods in the 1991–2018 period. For these analyses we considered the first publication containing new information regarding a treatment (either a Cochrane review or a clinical guideline) as the first landmark (File S2). Based on the year of publication of this landmark and that of the guidelines regarding a treatment, we specified three time‐periods in accordance with Roger's innovation theory indicating that behavioural change takes place in phases (Rogers, 2003): (I) from 1991 until year of the first landmark, (II) from the first landmark to five years after the guideline publication, and (III) from the end of period II until 2018. We included time‐period (i.e. I, II or III) as a categorical variable in the regression model and adjusted all analyses for age group, sex and their two‐way interaction. We report adjusted incidence rate ratios (IRR) with 95% confidence intervals, with confidence intervals based on robust estimators for the standard error.

We conducted a sensitivity analysis where diagnosis specific codes for spinal fusion and invasive pain treatment were assessed (i.e. stratifying by the diagnosis categories: non‐specific LBP, degenerative low back problems and spinal stenosis). We also assessed the robustness of our primary analyses with Poisson regression analyses using generalized estimating equations. The generalized estimating equation analysis used an independent working correlation structure and robust standard errors to account for the possible correlation of outcomes within same age group and sex stratum in different years. We conducted a third set of sensitivity analyses with the logarithm of the population size as offset added to the model to take population dynamics into consideration. We conducted all analyses in SPSS version 26.

### 2.5 Ethical considerations

As our data were collected for registration purposes and only contained non‐identifiable information, the Dutch Medical Research Involving Human Subjects Act does *not* apply to this study (as acknowledged by the Amsterdam UMC, location VUmc medical ethical committee, reference no. 2020.0665). As such, official approval by a medical ethical committee was not required and no informed consents were obtained.

## 3 RESULTS

**FIGURE 1:**
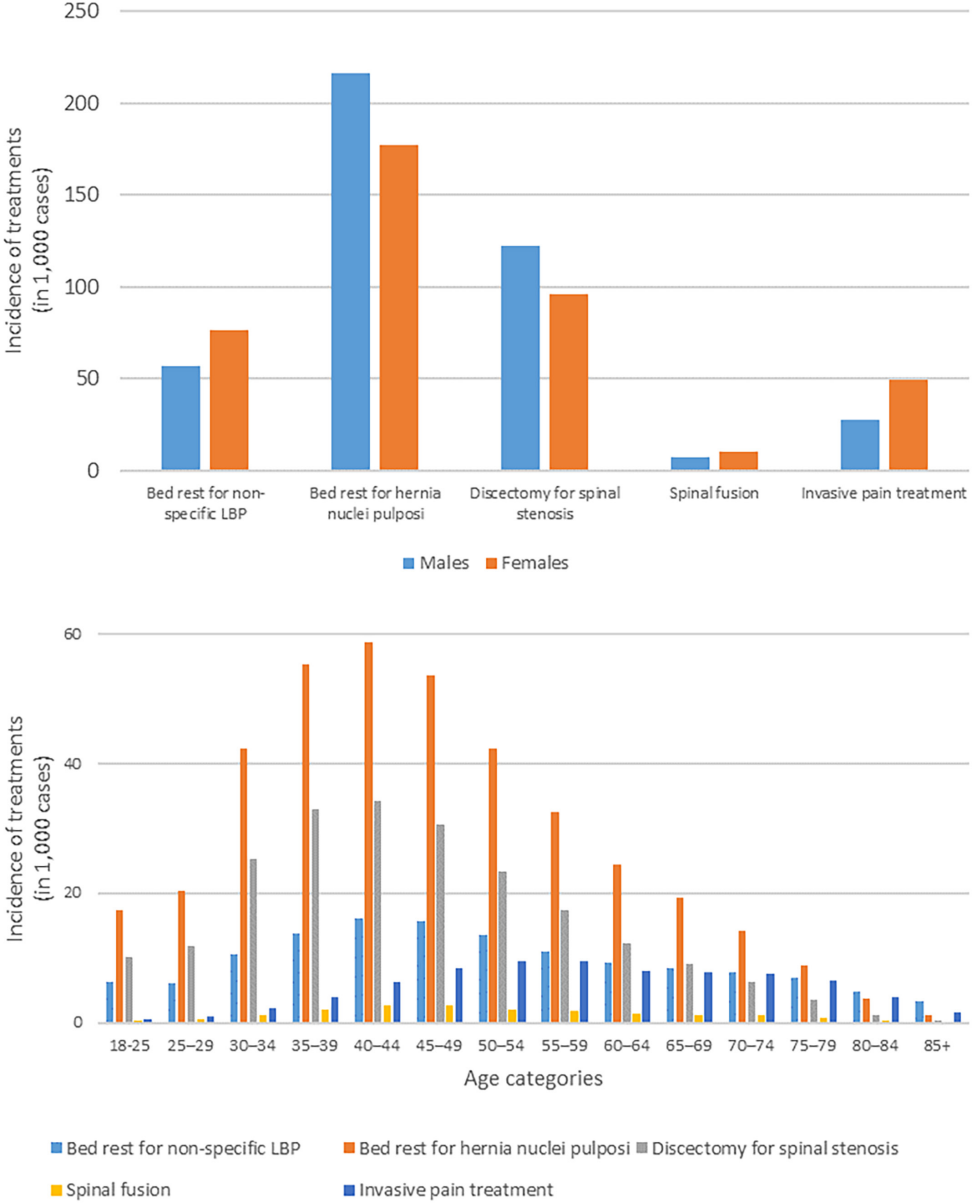
Use of absolute low back pain treatments, stratified by sex (upper panel) and age category (lower panel).

Figure 1 and File S6 show descriptive information of all studied LBP treatments. In the 1991–2018 period, there were 133,631 (37.6/100,000 inhabitants) treatments of bed rest for non‐specific LBP and 393,790 (110.8/100,000 inhabitants) treatments of bed rest for hernia nuclei pulposi. There were 218,635 (61.5/100,000 inhabitants) discectomies for spinal stenosis, 18,051 (1.7/100,000 inhabitants) spinal fusion surgeries and 77,259 (7.2/100,000 inhabitants) invasive pain treatments. Bed rest for non‐specific LBP, spinal fusion and invasive pain treatment were more common among females than among males (with 57%, 60% and 64% of these treatments being provided to females, respectively). The opposite was true for bed rest for hernia nuclei pulposi and discectomy for spinal stenosis (with respectively 45% and 45% of these treatments being provided to females). All treatments were most common in the 40–49‐year age group, except for invasive pain treatment which was most common among those aged 50–59 years. For relative treatments (expressed per 100,000 inhabitants), the age at which treatments were most common was higher (e.g. 80–84 years for bed rest for LBP and 75–79 for invasive pain treatment).

**FIGURE 2:**
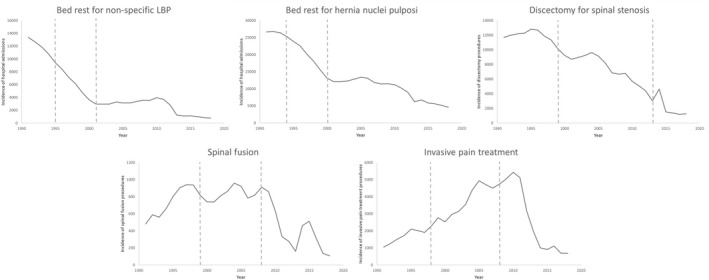
Time trends (i.e. yearly incidence) in the Netherlands in the time‐period 1991–2018 of the following treatments: Bed rest for LBP, bed rest for hernia nuclei pulposi, discectomy for spinal stenosis, and spinal fusion and invasive pain treatment for treatment for non‐specific LBP, degenerative low back problems and spinal stenosis. Vertical dashed lines depict the boundaries for the three time‐periods that are used for analyses.

Bed rest for non‐specific LBP and hernia nuclei pulposi and discectomy for spinal stenosis reduced gradually between 1991 and 2018; from 13,363 to 789 (91% de‐implementation since the first landmark publication), from 26,649 to 4608 (81% de‐implementation since the first landmark) and from 11,691 to 1259 (86% de‐implementation since the first landmark), respectively (Figure 2; File S7). For bed rest for non‐specific LBP and for discectomy, more than 84% de‐implementation took place after 18 and 17 years since the first landmark publication, respectively. For spinal fusion and invasive pain treatment there was an initial increase in treatments over time after which a reduction took place; for these treatments there was a reduction from 1991 to 2018 from 482 to 108 (85% de‐implementation since the first landmark, while 84% de‐implementation took place after 19 years) and from 1053 to 687 (75% de‐implementation since the first landmark), respectively. The initial increase in treatments was mainly seen for degenerative LBP and spinal stenosis, but not for non‐specific LBP (File S8). The reduction in spinal fusion and invasive pain treatments after 2008 was, however, seen across all diagnosis groups.

Trend‐analyses, depicting change in treatment use over time are shown in Table 1 (main analyses) and File S8 (for sensitivity analyses stratified on diagnosis). For bed rest for non‐specific LBP, we found statistically significant decreases in yearly use between period. IRRs were 0.47 (95% CI: 0.44–0.51) for period II compared to period I, that is, the yearly number of treatments reduced by 53%, and 0.46 (95% CI: 0.42–0.50) for period III compared to period I. For bed rest for hernia nuclei pulposi, IRRs were 0.72 (95% CI: 0.68–0.76) for period II compared to period I and 0.52 (95% CI: 0.49–0.55) for period III compared to period I.

**TABLE 1:** Poisson regression analyses showing trends in treatments

	Poisson GLM					Poisson GLM with population size as offset				
	Overall test	Period II vs I^a^		Period III vs II^a^		Overall test	Period II vs I^a^		Period III vs II^a^	
	*p*‐value	IRR (95% CI)^b^	*p*‐value	IRR (95% CI)^b^	*p*‐value	*p*‐value	IRR (95% CI)^c^	p‐value	IRR (95% CI)^c^	*p*‐value
Bed rest for non‐specific low back pain	<0.001	0.47 [0.44 0.51]	<0.001	0.46 [0.42 0.50]	<0.001	< 0.001	0.44 [0.41 0.48]	<0.001	0.42 [0.38 0.45]	<0.001
Bed rest for hernia nuclei pulposi	<0.001	0.72 [0.68 0.76]	<0.001	0.52 [0.49 0.55]	<0.001	< 0.001	0.68 [0.65 0.71]	<0.001	0.48 [0.46 0.51]	<0.001
Discectomy for spinal stenosis	<0.001	0.61 [0.58 0.64]	<0.001	0.28 [0.24 0.32]	<0.001	< 0.001	0.57 [0.54 0.59]	<0.001	0.27 [0.23 0.31]	<0.001
Spinal fusion	<0.001	1.13 [1.04 1.22]	0.005	0.45 [0.40 0.51]	<0.001	<0.001	1.02 [0.95 1.09]	1.0	0.43 [0.38 0.48]	<0.001
Invasive pain treatment	<0.001	2.21 [2.06 2.37]	<0.001	0.66 [0.59 0.74]	0.001	<0.001	1.94 [1.83 2.07]	<0.001	0.58 [0.52 0.66]	<0.001

Abbreviation: GLM, generalized linear model, with robust standard estimates.

^a^ Cells contain incidence rate ratios (IRR), their 95% confidence intervals and p‐values for comparing the incidences in the two periods. P‐values for comparing periods are Bonferroni corrected p‐values (corrected for two pairwise comparisons).

^b^ Adjusted for age group, sex and their two‐way interaction.

^c^ Adjusted for age group, sex and their two‐way interaction, with logarithm of population size used as offset.

We found statistically significant decreases in yearly use of discectomy for spinal stenosis between time‐periods, IRR of 0.61 (95% CI: 0.58–0.64) for period II compared to period I, and 0.28 (95% CI: 0.24–0.32) for period III compared to period I. Yearly use of lumbar fusion/laminectomies was higher in period II compared to period I (IRRs: 1.13 [95% CI: 1.04–1.22]), but decreased in period III compared to period I (IRR: 0.45 [95% CI: 0.40–0.51]). Yearly use of invasive pain treatment was higher in period II compared to period I, while there was a decrease in period III, IRRs 2.21 (95% CI: 2.06–2.37) for period II compared to period I, and 0.66 (95% CI: 0.59, 0.74) for period III compared to period I.

For all treatments, sensitivity analyses with generalized estimating equations showed comparable results, except for the yearly number of lumbar fusion/laminectomies no longer being statistically significantly different in period II and III compared to period I (File S8). When assessing relative use, findings were comparable to the main analyses except that they showed an attenuated effect for the difference between period I and II for spinal fusion (Table 1).

## 4 DISCUSSION

Using Dutch register data, we assessed de‐implementation of five hospital treatments for LBP that were no longer recommended in evidence and guidelines. Although incidence of all studied treatments reduced substantially between 1991 and 2018, the de‐implementation rate in Dutch hospitals was slow.

While de‐implementation was gradual for bed rest for non‐specific LBP and hernia nuclei pulposi and discectomy for spinal stenosis, by 2018 its use for these conditions had declined substantially by 91%, 81% and 86%, since the first landmark publication. De‐implementation beyond 84% was reached after 18 and 17 years for bed rest for non‐specific LBP and discectomy respectively, while this threshold had still not been reached at the end of the studied period for bed rest for hernia nuclei pulposi. These time lags are comparable or longer than the 17‐year time lag that was estimated to be needed for implementation of evidence into practice (Balas & Boren, 2000). It could be more difficult to de‐implementing a treatment already entrenched in routine care than implementing a new therapy.

For the other two treatments we reported an initial increase in use before their use declined. De‐implementation for spinal fusion was reached after 19 years, while invasive pain treatment did not reach 84% de‐implementation. A reason may be that, for these treatments, evidence preceded the guidelines instead of the other way around. This would be consistent with clinicians having difficulty keeping up with evidence (i.e. original scientific papers and/or reviews), while being more inclined to rely on guidelines to inform their practice (Penfield et al., 2014).

Our findings were fairly robust to sensitivity analyses using a different analytical approach and using relative rather than absolute numbers. However, for spinal fusion and invasive pain treatment, stratified analyses showed that de‐implementation predominantly took place for non‐specific LBP compared with degenerative LBP or spinal stenosis. This may be explained by differences in how the conditions and/or treatments are perceived, despite similar evidence and guidelines. Clinicians may consider a need to do ‘something’ for degenerative LBP or stenosis patients and consider for example, that invasive pain treatment may be preferable to bed rest, discectomy or fusion, even though evidence indicates all should be de‐implemented. Possibly because LBP patients desire patient‐centred care and to foster a relationship with their clinician (Chou et al., 2018), clinicians may consider ‘doing nothing’ not a viable option. This could be influenced by clinicians' perceptions of what patients want, patients' preferences (Schers et al., 2000), or beliefs and (lack of) knowledge of clinicians (Schers et al., 2001). The reported trends in our study show both similarities and differences with trends in the international context. For example, in contrast to our data, admissions for LBP (Anderson et al., 2022) and spinal fusions (Machado et al., 2017) increased rather than decreased between 2005 and 2013 in Australia. Also in the USA, spinal fusion increased in approximately that same time period (Martin et al., 2019). However, USA's invasive pain procedures increased between 2000 and 2008 after which, comparable to our Dutch data, a slight decline occurred (Manchikanti et al., 2020).

### 4.1 Implications for practice and policy

In the Dutch context, but probably also in other countries, there are two important drivers for (de‐)implementation of treatments. First, a professional association can advise, via guidelines, whether or not to provide treatments under certain circumstances. Guidelines are typically developed by guideline committees within professional associations, based on scientific evidence regarding (cost‐)effectiveness of treatments, but also considering experiences from practitioners and patients, and ethical and legal issues (Hulshof, 2009). Although guidelines are not legally binding, they provide a standard of care practitioners are expected to meet and have to justify when they deviate from them. After guideline publication, that we considered as landmarks in our analyses, a number of updated and/or additional guidelines were published (totalling 17 guidelines since 1995; File S1). These updated guidelines may have further accelerated de‐implementation of non‐evidence‐based care.

A second important driver for (de‐)implementation is healthcare reimbursement, in which clinicians are typically rewarded by volume and complex treatments rather than quality of care (Traeger et al., 2019). In the Netherlands, until 2006 health care was covered by private health insurers that provided a budget to health care facilities, based on the number of treatments given. In 2006, a basic health insurance scheme was introduced, which all Dutch inhabitants are obliged to accept. Under the new system, the government and health care facilities negotiate maximum prices for a certain diagnosis‐treatment combination. In 2012 this system further evolved to determine the price of treatments based on the desired outcome instead of the treatment. In 2015 the maximum price for each treatment‐diagnosis combination was reduced. All LBP treatments assessed in this study were covered by the Dutch basic health insurance between 1991 and 2018. Although the system has evolved over the years, putting less emphasis on volume of care, the reimbursement system could have delayed de‐implementation.

Other reasons for slow and incomplete de‐implementation include misconceptions regarding evidence and guidelines, fear of doing the wrong thing (Lin et al., 2018) and a desire to maintain good relationships with patients (Slade et al., 2015). The latter is in line with ‘shared decision making’ which is advocated in contemporary LBP guidelines, and may hinder complete de‐implementation of the assessed LBP treatments (Foster et al., 2018). The routine collection and availability of clinical data in registries (such as data from this study), can be enriched with patient reported outcome measures (PROMS), facilitating this shared decision making and thus improve future care.

Despite an evolution of system factors, such as guideline development and changes in the way that healthcare is funded, our study shows that de‐implementation rate is slow and non‐evidence‐based LBP treatments are still used in the Dutch healthcare system. Further adjustment of reimbursement systems for LBP treatments based on evidence and together with professional guidelines may accelerate de‐implementation. Moreover, only publication of guidelines may not be sufficiently effective, without accompanying them with appropriate implementation activities. Such activities can, for example, include easy to understand messages that can be conveyed in social‐ or mass‐media campaigns (Suman et al., 2021), or (post‐graduate) training, education, audit and feedback (Ivers et al., 2012) for clinicians and other relevant stakeholders. Researchers, together with stakeholders, could work on developing such activities to enhance de‐implementation of non‐evidence‐based LBP treatments.

### 4.2 Methodological considerations and research implications

We reported register data providing a realistic overview of the care in Dutch general and academic hospitals and some clinics over a 28‐year period. These historic data, however, come with the limitation that diagnosis and treatment categories were based on what was relevant in the early 90s, and may no longer be relevant today. For example, categories of non‐specific and degenerative LBP are separate entities in our registry, whereas the more contemporary way to approach LBP would be to consider these together as ‘non‐specific LBP’ (Maher et al., 2017). Moreover, no information on, for example, the duration and/or impact of the diseases or way of diagnoses are available in our data. While the focus of our study was on low‐back pain, the available data consists of some treatment codes referring to other areas of the spine (e.g. thermolysis at the cervical and thoracic level). Such codes would ideally have been excluded from our analyses, which unfortunately was not possible given the data structure.

Also, certain assumptions were made when determining the diagnosis and treatment categories. For example, bed rest was operationalized as hospital admissions, which may not be valid in all instances. Invasive pain treatment is a category of different treatment options, including injection therapy, percutaneous thermolysis, incision, dissection and adhesiolysis. Although guidelines advice against the use of all invasive pain treatments (CBO, 2003), evidence on the wide variety of treatments in this category is still limited. The most recent guideline, that was published in 2011, still advises to conduct invasive treatment only in well selected patient populations or under experimental conditions to gain more evidence on the topic (Ligtenberg et al., 2011). Moreover, there were some missing data in the period preceding 2014, with incompleteness ranging from 0.3% in 2002 to 18% in 2012. This could mean that for earlier study periods, we may have overestimated the true de‐implementation rate. Moreover, guidelines and the context of clinical care in the Netherlands may be different from those in other countries. It is thus unclear to what extent the results of our study can be extrapolated to other contexts and replication of our study in other countries and regions would be worthwhile.

We did not use a reference treatment (e.g. assessing use of different treatments during the same time‐period). This would have helped us to tease out effects caused by things other than guidelines or evidence, such as changes in reimbursement structures or changes in population demographics over time. It is likely, however, that any effects of these issues on our findings would have been small, given that the studied treatments were consistently reimbursed by health insurance during the 1991–2018 period and because effects remained consistent when analysing relative incidence.

The cut‐off points chosen for the time‐periods that we used in our additional supporting analyses were relatively arbitrary. Choosing a single time point from when de‐implementation can be expected, such as in earlier research (Balas & Boren, 2000), seems to oversimplify the issue as de‐implementation should be considered as a process over time (Rogers, 2003). Even the landmark of a publication of a guideline or Cochrane review is arbitrary as these landmarks are likely to be preceded by single study publications, or non‐Cochrane systematic reviews. Addressing these additional landmarks should be a focus of future research. Nonetheless choosing different time‐periods or cut‐off points would unlikely have led to different conclusions. It should be noted that the accumulation of evidence and guideline development is an ongoing process. Future evidence could therefore change the advices in guidelines for certain procedures or (sub‐)groups of patients or could even advocate the use of personalized medicine, in particular for those procedures for which we currently have limited evidence in the scientific literature. Moreover, research has shown that we must be careful with, for example, surgical and other invasive interventions for low‐back pain (Foster et al., 2018). The most recent guideline, that was published in 2011, still advises not to conduct invasive pain treatment in non‐specific low‐back pain patients and to restrict this to only strictly specific patient populations and/or patients in clinical studies (Ligtenberg et al., 2011).

We quantified de‐implementation of hospital LBP treatments by assessing their use in Dutch hospitals over time. De‐implementation for individual practitioners or other settings (e.g. outpatient care) may be different, and this could be a focus for future studies. The majority of Dutch LBP patients are likely to visit primary care (e.g. general practitioners or physiotherapists), where they will be treated or referred to secondary care (e.g. hospitals) for additional diagnosis and/or treatment. Although the hospital data also reflect the referral behaviour of primary care, it would be useful for future studies to address the de‐implementation of LBP treatments also specifically in primary care.

## 5 CONCLUSION

De‐implementation of five non‐evidence‐based Dutch hospital treatments for LBP was substantial but took several decades. This has likely resulted in considerable waste of resources and avoidable harm to many patients. More attention is needed for effective strategies to accelerate de‐implementation of non‐evidence‐based LBP care, while simultaneously working towards better (sub‐)diagnoses, and updating treatment descriptions.

## AUTHOR CONTRIBUTIONS

JA conceptualized the project. PvdV analysed the data. PC and AdW drafted the manuscript. All authors (PC, AdW, PvdV, RB, MdM, BK, JH and JA) reviewed the manuscript for important intellectual content. JA is the study guarantor.

## FUNDING INFORMATION

There was no particular funding for this project.

## CONFLICTS OF INTERESTS

BK was part of teams developing some of the guidelines mentioned in this paper. Otherwise, there are no conflicts of interest declared by the authors.

## Supporting information


Supplementary file S1.

Supplementary file S2

Supplementary file S3

Supplementary file S4

Supplementary file 5

Supplementary file S6

Supplementary file S7

Supplementary file S8
