# Supplementary material for: The slow de‐implementation of non‐evidence‐based treatments in low back pain hospital care—Trends in treatments using Dutch hospital register data from 1991 to 2018
Source: Eur J Pain. 2022 Nov 12;27(2):212–22. doi: 10.1002/ejp.2052 (PMC10099564; doi:10.1002/ejp.2052)
Supplement: Supplementary file 1 — Supplementary file S1. [file EJP-27-212-s008.pdf]

Supplementary file 1. Systematic search for Dutch guidelines on low-back pain.

Selection of guidelines

Guidelines on low-back pain were searched in the biblical databases PubMed, EMBASE, CINAHL, Cochrane and Web of Science (from database inception to April 2018). Search terms and strategy are shown below. In addition the internet was searched on two Dutch guideline databases and websites of health care practitioner professional organizations/boards (also shown in the below). Finally personal communication with guideline developers was used to identify all versions of a guideline (original version and all updates). Abstracts and titles of identified records were screened for eligibility by two authors independently using the following inclusion and exclusion criteria.

Inclusion criteria:

- Patients with low-back pain are the primary target group of the guideline;
- Guideline is issued and authorized by professional heath care practitioner organizations/boards;
- Original guidelines and revised versions;
- Guideline could target health care practitioners in primary and/or secondary care;
- Guideline could be multi- or mono-disciplinary;

Exclusion criteria:

- Guidelines with copied, summarized or translated content from original practice guidelines
- Guidelines for health care professionals not involved in care for low-back pain

Results

The flowchart below shows the selection procedure of guidelines. We identified 513 records in biblical databases and 33 records identified on guideline databases and websites of health care practitioner professional organizations/boards. After removing duplicates, 254 records were screened on title and abstract, from which 213 were excluded. The remaining 23 records were screened to identify Dutch back pain guidelines. After reading the full texts of those guidelines, 6 were excluded, because these guidelines were not original but summaries of other guidelines (4), book chapters (1) or not supported/issued by professional associations of health care professionals (1). Seventeen health care practitioner guidelines (including updated versions) published since 1995 were included:

- Kwaliteitsinstituut voor de Gezondheidszorg (CBO), multi-disciplinary guideline; 1995
- Kwaliteitsinstituut voor de Gezondheidszorg (CBO), multi-disciplinary guideline; 2003
- Kwaliteitsinstituut voor de Gezondheidszorg (CBO), multi-disciplinary guideline; 2010
- Neurology guidelines; 2008
- Koninklijk Nederlands Genootschap voor Fysiotherapie (KNGF), physiotherapy guideline; 2001
- Koninklijk Nederlands Genootschap voor Fysiotherapie (KNGF), physiotherapy guideline; 2005
- Koninklijk Nederlands Genootschap voor Fysiotherapie (KNGF), physiotherapy guideline; 2013
- Nederlands Huisartsen Genootschap (NHG), general practitioner guideline non-specific low-back pain; 1996
- Nederlands Huisartsen Genootschap (NHG), general practitioner guideline non-specific low-back pain; 2005
- Nederlands Huisartsen Genootschap (NHG), general practitioner guideline non-specific low-back pain; 2015
- Nederlands Huisartsen Genootschap (NHG), general practitioner guideline lumbosacral radicular syndrome; 1996
- Nederlands Huisartsen Genootschap (NHG), general practitioner guideline lumbosacral radicular syndrome; 2005
- Nederlands Huisartsen Genootschap (NHG), general practitioner guideline lumbosacral radicular syndrome; 2015
- Nederlandse Vereniging voor Arbeids- en Bedrijfsgeneeskunde (NVAB), guideline for occupational physicians; 1999
- Nederlandse Vereniging voor Arbeids- en Bedrijfsgeneeskunde (NVAB), guideline for occupational physicians; 2006
- Nederlandse Orthopaedische Vereniging (NOV), guideline for orthopaedic surgeons; 2011
- Vereniging van Oefentherapeuten Cesar en Mensendieck (VvOCM), guideline for exercise and Mensendieck therapy 2009

Flow chart depicting the guideline selection procedure

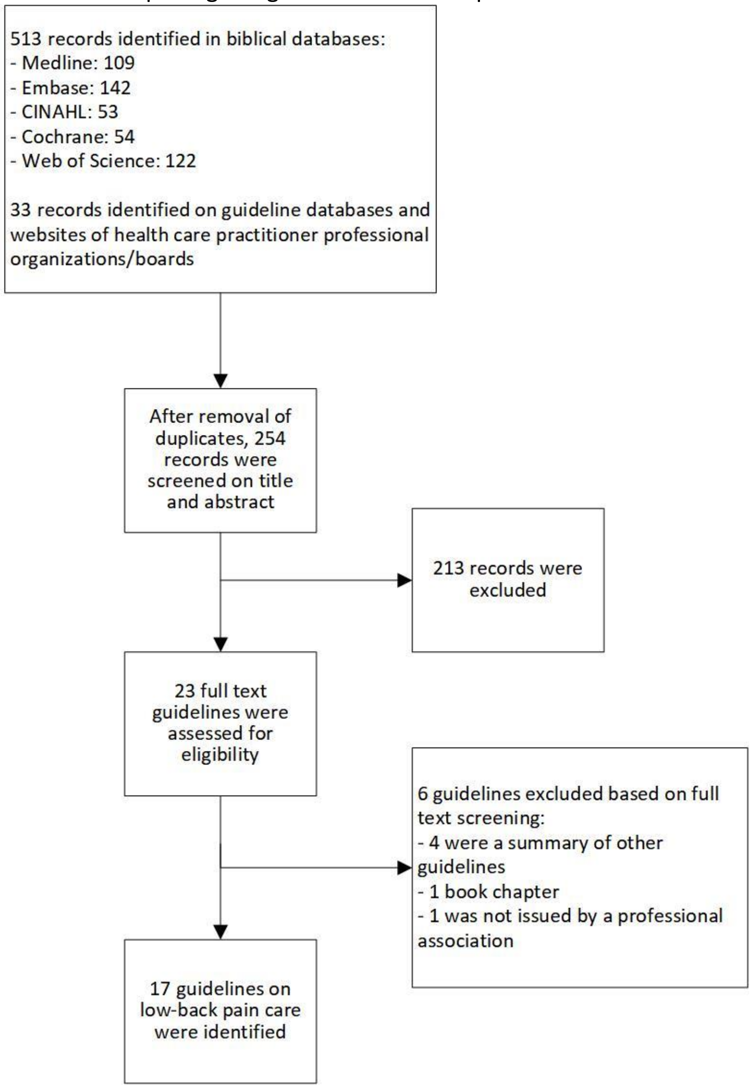

Search strategy in electronic biblical databases

Electronic search in Medline

Search	Query	Results
#9	Search #7 AND #8	109
#8	Search "Netherlands"[Mesh] OR netherland*[tiab] OR dutch[tiab] OR holland[tiab]	93368
#7	Search #5 AND #6	3946
#6	Search (("Guideline" [Publication Type] OR "Guidelines as Topic"[Mesh:NoExp] OR "Practice Guidelines as Topic"[Mesh] OR "Guideline Adherence"[Mesh])) OR guideline*[tiab] OR "Clinical Protocols"[Mesh:NoExp] OR protocol*[tiab]	740533
#5	Search #2 OR #3 OR #4	85027
#4	Search "Intervertebral Disc"[Mesh] OR "Intervertebral Disc Degeneration"[Mesh] OR "Intervertebral Disc Displacement"[Mesh] OR "Intervertebral Disc Chemolysis"[Mesh] OR "Intervertebral disc disease" [Supplementary Concept] OR Intervertebral Dis*[tiab] OR Annulus Fibrosus[tiab] OR Nucleus Pulposus[tiab] OR Disc Degeneration*[tiab] OR Disk Degeneration*[tiab] OR Discolysis[tiab] OR Discolyses[tiab] OR Diskolysis[tiab] OR Diskolyses[tiab] OR Chemonucleolysis[tiab] OR Chemonucleolyses[tiab] OR Lumbar Disc Disease*[tiab] OR Lumbar Disk Disease*[tiab] OR Disc Prolapse*[tiab] OR Disk Prolapse*[tiab] OR Herniated Disc*[tiab] OR Herniated Disk*[tiab] OR Prolapsed Disc*[tiab] OR Prolapsed Disk*[tiab] OR Slipped Disc*[tiab] OR Slipped Disk*[tiab]	34587

#3	Search ("Radiculopathy"[Mesh] OR radiculopath*[tiab]) AND ("Lumbosacral region"[Mesh] OR "Lumbar Vertebrae"[Mesh] OR lumbar[tiab] OR lumbosacral[tiab])	3539
#2	Search ("Back Pain"[Mesh:NoExp]) OR "Low Back Pain"[Mesh] OR (back pain[tiab] OR back pains[tiab]) OR (back ache[tiab] OR back aches[tiab]) OR (backache[tiab] OR backache'[tiab] OR backaches[tiab]) OR (lumbago[tiab] OR lumbago'[tiab] OR lumbagoes[tiab] OR lumbagoischias[tiab] OR lumbagos[tiab] OR lumbagosciatica[tiab]) OR (vertebrogenic pain syndrome[tiab] OR vertebrogenic pain syndromes[tiab])	55848

Electronic search in Embase

Search	Query	Results
#8	#6 AND #7	142
#7	'netherlands'/exp OR netherland*:ti,ab,kw OR dutch:ti,ab,kw OR holland:ti,ab,kw	119801
#6	#4 AND #5	7924
#5	'practice guideline'/exp OR 'guideline'/exp OR 'protocol'/exp OR 'protocol compliance'/exp OR guideline*:ti,ab,kw OR 'clinical protocol'/exp OR protocol*:ti,ab,kw	1164658
#4	#1 OR #2 OR #3	143338
#3	'intervertebral disk hernia'/exp OR 'intervertebral disk degeneration'/exp OR 'chemonucleolysis'/exp OR 'intervertebral disk disease'/exp OR 'intervertebral dis*':ti,ab,kw OR 'annulus fibrosus':ti,ab,kw OR 'nucleus pulposus':ti,ab,kw OR 'disc degeneration*':ti,ab,kw OR 'disk degeneration*':ti,ab,kw OR discolysis:ti,ab,kw OR discolyses:ti,ab,kw OR diskolysis:ti,ab,kw OR diskolyses:ti,ab,kw OR chemonucleolysis:ti,ab,kw OR chemonucleolyses:ti,ab,kw OR 'lumbar disc disease*':ti,ab,kw OR 'lumbar disk disease*':ti,ab,kw OR 'disc prolapse*':ti,ab,kw OR 'disk prolapse*':ti,ab,kw OR 'herniated disc*':ti,ab,kw OR 'herniated disk*':ti,ab,kw OR 'prolapsed disc*':ti,ab,kw OR 'prolapsed disk*':ti,ab,kw OR 'slipped disc*':ti,ab,kw OR 'slipped disk*':ti,ab,kw	46300
#2	('radiculopathy'/exp OR radiculopath*:ti,ab,kw) AND ('lumbosacral region'/exp OR 'lumbar vertebra'/exp OR lumbar:ti,ab,kw OR lumbosacral:ti,ab,kw)	7443
#1	'backache'/de OR 'discogenic pain'/exp OR 'low back pain'/exp OR 'back pain*':ti,ab,kw OR 'back ache*':ti,ab,kw OR backache*:ti,ab,kw OR lumbago*:ti,ab,kw OR 'vertebrogenic pain syndrome*':ti,ab,kw	106206

Electronic search in CINAHL

Search	Query	Results
S8	S6 AND S7	53
S7	(MH "Netherlands") OR TI (netherland* OR dutch OR Holland) OR AB (netherland* OR dutch OR Holland)	20,762
S6	S4 AND S5	1,435
S5	(MH "Guideline Adherence") OR (MH "Protocols") OR TI (guideline* OR protocol*) OR AB (guideline* OR protocol*)	123,857
S4	S1 OR S2 OR S3	25,803
S3	(MH "Intervertebral Disk+") OR (MH "Intervertebral Disk Chemolysis") OR (MH "Intervertebral Disk Displacement") OR TI (“Intervertebral Dis*” OR “Annulus Fibrosus” OR “Nucleus Pulposus” OR “Disc Degeneration*” OR “Disk Degeneration*” OR Discolysis OR Discolyses OR Diskolysis OR Diskolyses OR Chemonucleolysis OR Chemonucleolyses OR “Lumbar Disc Disease*” OR “Lumbar Disk Disease*” OR “Disc Prolapse*” OR “Disk Prolapse*” OR “Herniated Disc*” OR “Herniated Disk*” OR “Prolapsed Disc*” OR “Prolapsed Disk*” OR “Slipped Disc*” OR “Slipped Disk*”) OR AB (“Intervertebral Dis*” OR “Annulus Fibrosus” OR “Nucleus Pulposus” OR “Disc Degeneration*” OR “Disk Degeneration*” OR Discolysis OR Discolyses OR Diskolysis OR Diskolyses OR Chemonucleolysis OR Chemonucleolyses OR “Lumbar Disc Disease*” OR “Lumbar Disk Disease*” OR “Disc Prolapse*” OR “Disk Prolapse*” OR “Herniated Disc*” OR “Herniated Disk*” OR “Prolapsed Disc*” OR “Prolapsed Disk*” OR “Slipped Disc*” OR “Slipped Disk*”)	4,294

S2	((MH "Radiculopathy") OR TI (radiculopath*) OR AB (radiculopath*)) AND ((MH "Lumbar Vertebrae") OR (TI (lumbar OR lumbosacral)) OR (AB (lumbar OR lumbosacral)))	629
S1	(MH "Back Pain") OR (MH "Low Back Pain") OR TI (“back pain*” OR “back ache*” OR backache* OR lumbago* OR “vertebrogenic pain syndrome*”) OR AB (“back pain*” OR “back ache*” OR backache* OR lumbago* OR “vertebrogenic pain syndrome*”)	22,671

Electronic search in Cochrane Library

Search	Query	Results
#8	#6 and #7	54
#7	guideline*:ti,ab,kw or protocol*:ti,ab,kw	92121
#6	#4 and #5	162
#5	netherland*:ti,ab,kw or dutch:ti,ab,kw or holland:ti,ab,kw	9561
#4	#1 or #2 or #3	12773
#3	"Intervertebral Dis*":ti,ab,kw or "Annulus Fibrosus":ti,ab,kw or "Nucleus Pulposus":ti,ab,kw or "Disc Degeneration*":ti,ab,kw or "Disk Degeneration*":ti,ab,kw or Discolysis:ti,ab,kw or Discolyses:ti,ab,kw or Diskolysis:ti,ab,kw or Diskolyses:ti,ab,kw or Chemonucleolysis:ti,ab,kw or Chemonucleolyses:ti,ab,kw or "Lumbar Disc Disease*":ti,ab,kw or "Lumbar Disk Disease*":ti,ab,kw or "Disc Prolapse*":ti,ab,kw or "Disk Prolapse*":ti,ab,kw or "Herniated Disc*":ti,ab,kw or "Herniated Disk*":ti,ab,kw or "Prolapsed Disc*":ti,ab,kw or "Prolapsed Disk*":ti,ab,kw or "Slipped Disc*":ti,ab,kw or "Slipped Disk*":ti,ab,kw	2361
#2	radiculopath*:ti,ab,kw and (lumbar:ti,ab,kw or lumbosacral:ti,ab,kw)	315
#1	"back pain*":ti,ab,kw or "back ache*":ti,ab,kw or backache*:ti,ab,kw or lumbago*:ti,ab,kw or "vertebrogenic pain syndrome*":ti,ab,kw	11099

Electronic search in Web of Science

Search	Query	Results
# 8	#7 AND #6	122
# 7	TOPIC: ((netherland* OR dutch OR holland))	132,065
# 6	#5 AND #4	5,246
# 5	TOPIC: ((guideline* OR protocol*))	813,390
# 4	#3 OR #2 OR #1	71,797
# 3	TOPIC: ((“Intervertebral Dis*” OR “Annulus Fibrosus” OR “Nucleus Pulposus” OR “Disc Degeneration*” OR “Disk Degeneration*” OR Discolysis OR Discolyses OR Diskolysis OR Diskolyses OR Chemonucleolysis OR Chemonucleolyses OR “Lumbar Disc Disease*” OR “Lumbar Disk Disease*” OR “Disc Prolapse*” OR “Disk Prolapse*” OR “Herniated Disc*” OR “Herniated Disk*” OR “Prolapsed Disc*” OR “Prolapsed Disk*” OR “Slipped Disc*” OR “Slipped Disk*”))	18,827
# 2	TOPIC: (((radiculopath*) AND (lumbar OR lumbosacral)))	2,572
# 1	TOPIC: ((“back pain*” OR “back ache*” OR backache* OR lumbago* OR “vertebrogenic pain syndrome*”))	57,180

Search for guidelines on two Dutch guideline databases and websites of health care practitioner professional organizations/boards.
Guideline databases:

- https://www.artsenapotheker.nl/huisarts/zoeken.html?query=rugpijn&dossierId=0&x=0&y=0&offset=2
- https://richtlijnendatabase.nl/?query=rug&specialism=&sort=1

Websites of health care practitioner professional organizations/boards:

- https://www.bivt.nl/pages/beroepsverenigingen/
- http://www.b9.nl/beroepsverenigingen/medisch.htm
- http://www.b9.nl/beroepsverenigingen/index.htm
- http://www.med-info.nl/Portal_Beroepsverenigingen.html
