# Supplementary material for: The slow de‐implementation of non‐evidence‐based treatments in low back pain hospital care—Trends in treatments using Dutch hospital register data from 1991 to 2018
Source: Eur J Pain. 2022 Nov 12;27(2):212–22. doi: 10.1002/ejp.2052 (PMC10099564; doi:10.1002/ejp.2052)
Supplement: Supplementary file 2 — Supplementary file S2 [file EJP-27-212-s004.pdf]

Supplementary file 2. Overview of Cochrane reviews indicating lacking evidence or evidence for marginal, no beneficial or even harmful effects for the treatment options: 1) bed rest for non-specific LBP and 2) bed rest for hernia nuclei pulposi, 3) discectomy for spinal stenosis, 4) spinal fusion and 5) invasive pain treatment for non-specific LBP, degenerative low back problems and spinal stenosis. Corresponding published guidelines in general advocate for more conservative treatment options.

**Treatments**	**Publication of Cochrane evidence**	**Summary of Cochrane evidence**	**Publication of the first guideline**	**Advice extracted from guidelines**	**Time-periods[1]**
Bed rest for non-specific LBP	Cochrane review in 2002(Hilde et al., 2002)	Small beneficial effects for the advice to stay active compared to bed rest.	General practitioner guideline 1996(Faas et al., 1996)	Bed rest is not advised for non-specific LBP.	I. 1991-1995<br>II. 1996-2001<br>III. 2002-2018
Bed rest for hernia nuclei pulposi	Cochrane review in 2002(Hilde et al., 2002)	Marginal differences between the advice to stay active compared to bed rest.	Multidisciplinary guideline (CBO) 1995(CBO, 1995)	Bed rest is not advised for hernia nuclei pulposi.	I. 1991-1994<br>II. 1995-2000<br>III. 2001-2018
Discectomy for lumbar spinal stenosis	Cochrane review in 2000(Gibson, Grant, et al., 2000)	Compared to conservative treatment, discectomy among carefully selected patients provides faster acute relief. Long-term effects are unclear.	Multidisciplinary guideline, 2008(CBO, 2008)	Conservative treatment (rather than discectomy) is advised for spinal stenosis.	I. 1991-1999<br>II. 2000-2013<br>III. 2014-2018
Lumbar fusion/laminectomy for degenerative low back problems/lumbar spinal stenosis/non-specific LBP	Cochrane review in 2000(Gibson, Waddell, et al., 2000)	No scientific evidence for the effectiveness of any form of surgical decompression or fusion for degenerative lumbar spondylosis compared with natural history, placebo, or conservative treatment	Multidisciplinary guideline, for non-specific LBP 2003(CBO, 2003)	No indication for surgery for a-specific LBP.	I. 1991-1999<br>II. 2000-2008<br>III. 2009-2018
Invasive pain treatment for degenerative low back problems/lumbar spinal stenosis/non-specific LBP	Cochrane review in 1999(Nelemans et al., 1999)	Insufficient evidence to support the use of injection therapy, regardless of type and dosage, for patients with subacute and chronic LBP without radicular pain	Multidisciplinary guideline, for non-specific LBP 2003(CBO, 2003)	Injection therapy is not advised for acute or chronic LBP.	I. 1991-1998<br>II. 1999-2008<br>III. 2009-2018

LBP = Low back pain

[1] Using the following time-periods: I) period from 1991 until year of the first landmark, II) period which starts with the first landmark and ends five years after the guideline publication, and III) period after period II until 2018.

References

CBO. (1995). *[Lumbosacraal radiculair syndroom, richtlijn CBO] Lumbosacral radicular syndrome, guideline CBO*.

CBO. (2003). *[Aspecifieke lage rugklachten] Non-specific low back pain*.

CBO. (2008). *[Richtlijn lumbosacraal radiculair syndroom] Guideline for lumbosacral radicular syndrom*.

Faas, A., Chavannes, A. W., Koes, B. W., van den Hoogen, J. M. M., Mens, J. M. A., Smeele, L. J. M., Romeijnders, A. C. M., & van der Laan, J. R. (1996). [NHG-standaard Lage-Rugpijn] NHG guideline for low back pain. *Huisarts en Wetenschap*, *39*(1), 18-31.

Gibson, J. N., Grant, I. C., & Waddell, G. (2000). Surgery for lumbar disc prolapse. *Cochrane Database of Systematic Reviews*, *3*, CD001350.

Gibson, J. N., Waddell, G., & Grant, I. C. (2000). Surgery for degenerative lumbar spondylosis. *Cochrane Database of Systematic Reviews*, *3*, CD001352.

Hilde, G., Hagen, K. B., Jamtvedt, G., & Winnem, M. (2002). Advice to stay active as a single treatment for low back pain and sciatica. *Cochrane Database of Systematic Reviews*, *2*, CD003632.

Nelemans, P., de Bie, R., de Vet, H. C. W., & Sturmans, F. (1999). Injection therapy for subacute and chronic benign low-back pain. *Cochrane Database of Systematic Reviews*, *4*, CD001824.
