# Supplementary material for: The slow de‐implementation of non‐evidence‐based treatments in low back pain hospital care—Trends in treatments using Dutch hospital register data from 1991 to 2018
Source: Eur J Pain. 2022 Nov 12;27(2):212–22. doi: 10.1002/ejp.2052 (PMC10099564; doi:10.1002/ejp.2052)
Supplement: Supplementary file 3 — Supplementary file S3 [file EJP-27-212-s001.pdf]

Supplementary file 3. Overview of incompleteness of the data for the variable ‘hospital admissions’

**Year**	**% incomplete**
1991	0.6
1992	0.7
1993	0.7
1994	0.7
1995	0.8
1996	0.8
1997	0.6
1998	0.7
1999	0.5
2000	0.8
2001	0.8
2002	0.3
2003	0.5
2004	1.1
2005	2.9
2006	8.9
2007	10.9
2008	11.7
2009	12.0
2010	10.9
2011	15.1
2012	18.0
2013	15.7
2014	1.2
2015	0.3
2016	0.0
2017	0.0
2018	0.0
2019	0.0
