# Supplementary material for: The slow de‐implementation of non‐evidence‐based treatments in low back pain hospital care—Trends in treatments using Dutch hospital register data from 1991 to 2018
Source: Eur J Pain. 2022 Nov 12;27(2):212–22. doi: 10.1002/ejp.2052 (PMC10099564; doi:10.1002/ejp.2052)
Supplement: Supplementary file 4 — Supplementary file S4 [file EJP-27-212-s002.pdf]

Supplementary file 4. Specification of ICD 9 and 10 codes that are included in the four low back pain category

Low back pain category	ICD-9	ICD-10
Non-specific low back pain	722.30	M51.4
Non-specific low back pain	722.31	M51.4
Non-specific low back pain	722.32	M51.4
Non-specific low back pain	722.39	M51.4
Non-specific low back pain	724.2	M54.56
Non-specific low back pain	724.3	M54.39
Non-specific low back pain	724.4	M54.19
Non-specific low back pain	724.5	M54.99
Non-specific low back pain	724.6	M53.88
Non-specific low back pain	724.8	M54.89
Non-specific low back pain	724.9	M53.99
Non-specific low back pain	739.3	M99.03
Non-specific low back pain	739.4	M99.04
Degenerative low back problems	721.3	M47.87
Degenerative low back problems	721.5	M48.29
Degenerative low back problems	721.6	M48.19
Degenerative low back problems	721.7	M48.39
Degenerative low back problems	721.8	M48.89
Degenerative low back problems	721.90	M47.99
Degenerative low back problems	721.91	M47.19
Degenerative low back problems	722.6	M51.3
Degenerative low back problems	722.90	M51.9
Degenerative low back problems	722.91	M50.9
Degenerative low back problems	722.92	M51.9
Degenerative low back problems	722.93	M51.9
Lumbar spinal stenosis	721.41	M47.14
Lumbar spinal stenosis	721.42	M47.16
Lumbar spinal stenosis	724.00	M48.09
Lumbar spinal stenosis	724.01	M48.04
Lumbar spinal stenosis	724.02	M48.06
Lumbar spinal stenosis	724.09	M48.08
Hernia nuclei pulposi	722.10	M51.2
Hernia nuclei pulposi	722.11	M51.2
Hernia nuclei pulposi	722.2	M51.2
Hernia nuclei pulposi	722.70	M51.0
Hernia nuclei pulposi	722.71	M50.0
Hernia nuclei pulposi	722.72	M51.0
Hernia nuclei pulposi	722.73	M51.0
