# Supplementary material for: The slow de‐implementation of non‐evidence‐based treatments in low back pain hospital care—Trends in treatments using Dutch hospital register data from 1991 to 2018
Source: Eur J Pain. 2022 Nov 12;27(2):212–22. doi: 10.1002/ejp.2052 (PMC10099564; doi:10.1002/ejp.2052)
Supplement: Supplementary file 5 [file EJP-27-212-s007.pdf]

Supplementary file 5. Specification of treatments codes (in Dutch: Classificatie van Verrichtingen; CVV) from the operations thesaurus that are included in the four low back pain categories.

**Low back treatments**	**CVV group**	**CVV code**	**Description (Dutch)**	**Description (English)**
Discectomy incl. chemonucleolysis	5803	5803	Excisie en destructie van discus intervertebralis	Excision and destruction of the discus intervertebralis
Discectomy incl. chemonucleolysis	5803	58030	Chemonucleolyse en thermolaesie	Chemonucleolysis and thermolysis
Discectomy incl. chemonucleolysis	5803	580300	Chemonucleolyse of thermolaesie, nno	Chemonucleolysis or thermolysis, not specified
Discectomy incl. chemonucleolysis	5803	580301	Chemonucleolyse of thermolaesie, cervicaal	chemonucleolysis or thermolysis, cervical
Discectomy incl. chemonucleolysis	5803	580302	Chemonucleolyse of thermolaesie, thoracaal	chemonucleolysis or thermolysis, thoracal
Discectomy incl. chemonucleolysis	5803	580303	Chemonucleolyse of thermolaesie, lumbaal	chemonucleolysis or thermolysis, lumbal
Discectomy incl. chemonucleolysis	5803	58031	excisie van discus intervertebralis, cervicaal, anterior	Excision of the discus intervertebralis, cervical, anterior
Discectomy incl. chemonucleolysis	5803	58032	Excisie van discus intervertebralis, cervicaal, lateraal	Excision of the discus intervertebralis, cervical, lateral
Discectomy incl. chemonucleolysis	5803	58033	Excisie van discus intervertebralis, thoracaal	Excision of the discus intervertebralis, thoracal
Discectomy incl. chemonucleolysis	5803	58034	Excisie van discus intervertebralis, lumbaal	Excision of the discus intervertebralis, lumbal
Discectomy incl. chemonucleolysis	5803	580340	Excisie van discus intervertebralis, lumbaal, nno	Excision of the discus intervertebralis, lumbal, not specified
Discectomy incl. chemonucleolysis	5803	580341	Excisie van discus intervertebralis, lumbaal m.b.v. endoscopie	Excision of the discus intervertebralis, lumbal using endoscopy
Discectomy incl. chemonucleolysis	5803	580342	Excisie van discus intervertebralis, lumbaal, open	Excision of the discus intervertebralis, lumbal, open
Discectomy incl. chemonucleolysis	5803	58035	Excisie van discus intervertebralis, lumbaal, recidief	Excision of the discus intervertebralis, lumbal, relapse
Discectomy incl. chemonucleolysis	5803	58036	Excisie van overige discus intervertebralis, recidief	Excision of other discus intervertebralis, relapse
Discectomy incl. chemonucleolysis	5803	58037	Percutane discectomie	percutaneous discectomy
Discectomy incl. chemonucleolysis	5803	58038	Overige gespecificeerde excisie en destructie van discus intervertebralis	other specified excision and destruction of the discus intervertebralis
Discectomy incl. chemonucleolysis	5803	58039	Excisie en destructie van discus intervertebralis, niet gespecificeerd	Excision and destruction of the discus intervertebralis, non-specific
Laminectomy	5030	5030	Exploratie en decompressie van ruggenmergkanaal	Exploration and decompression of the spinal canal
Laminectomy	5030	50300	Laminectomie, cervicaal	laminectomy, cervical
Laminectomy	5030	503000	Laminectomie, cervicaal, nno	laminectomy, cervical, not specified
Laminectomy	5030	503001	Laminectomie, cervicaal, m.b.v. endoscopie	laminectomy, cervical, using endoscopy
Laminectomy	5030	503002	Laminectomie, cervicaal, open	laminectomy, cervical, open

Laminectomy	5030	50301	Laminectomie, thoracaal	laminectomy, thoracal
Laminectomy	5030	503010	Laminectomie, thoracaal, nno	laminectomy, thoracal, not specified
Laminectomy	5030	503011	Laminectomie, thoracaal, m.b.v. endoscopie	laminectomy, thoracal, using endoscopy
Laminectomy	5030	503012	Laminectomie, thoracaal, open	laminectomy, thoracal, open
Laminectomy	5030	50302	Laminectomie, lumbaal	laminectomy, lumbal
Laminectomy	5030	503020	Laminectomie, lumbaal, nno	laminectomy, lumbal, not specified
Laminectomy	5030	503021	Laminectomie, lumbaal, m.b.v. endoscopie	laminectomy, lumbal, using endoscopy
Laminectomy	5030	503022	Laminectomie, lumbaal, open	laminectomy, lumbal, open
Laminectomy	5030	50303	Relaminectomie, cervicaal	relaminectomy, cervical
Laminectomy	5030	503030	Relaminectomie, cervicaal, nno	relaminectomy, cervical, not specified
Laminectomy	5030	503031	Relaminectomie, cervicaal, m.b.v. endoscopie	relaminectomy, cervical, using endoscopy
Laminectomy	5030	503032	Relaminectomie, cervicaal, open	relaminectomy, cervical, open
Laminectomy	5030	50304	relaminectomie, thoracaal	relaminectomy, thoracal
Laminectomy	5030	503040	relaminectomie, thoracaal, nno	relaminectomy, thoracal, not specified
Laminectomy	5030	503041	relaminectomie, thoracaal, m.b.v. endoscopie	relaminectomy, thoracal, using endoscopy
Laminectomy	5030	503042	relaminectomie, thoracaal, open	relaminectomy, thoracal, open
Laminectomy	5030	50305	relaminectomie, lumbaal	relaminectomy, lumbal
Laminectomy	5030	503050	relaminectomie, lumbaal, nno	relaminectomy, lumbal, not specified
Laminectomy	5030	503051	relaminectomie, lumbaal, m.b.v. endoscopie	relaminectomy, lumbal, using endoscopy
Laminectomy	5030	503052	relaminectomie, lumbaal, open	relaminectomy, lumbal, open
Laminectomy	5030	50306	herstel van intraspinale gevolgen van wervelfractuur	recovery of intraspinal effects of spinal fracture
Laminectomy	5030	50308	overige gespecificeerde exploratie en decompressie van ruggenmergkanaal	other specified exploration and decompression of the spinal canal
Laminectomy	5030	50309	exploratie en decompressie van ruggenmergkanaal, niet gespecificeerd	exploration and decompression of the spinal canal, non-specific
Invasive pain treatment	5031	5031	Incisie, doorsnijden of destructie van intraspinale zenuwwortel of spinaal ganglion	Incision, dissection or destruction of the intraspinal nerve root or spinal ganglion
Invasive pain treatment	5031	50310	percutane thermolaesie van spinaal ganglion	percutaneous thermolysis of the spinal ganglion
Invasive pain treatment	5031	503100	percutane thermolaesie van spinaal ganglion, nno	percutaneous thermolysis of the spinal ganglion, not specified
Invasive pain treatment	5031	503101	percutane thermolaesie van spinaal ganglion, cervicaal	percutaneous thermolysis of the spinal ganglion, cervical
Invasive pain treatment	5031	503102	percutane thermolaesie van spinaal ganglion, thoracaal	percutaneous thermolysis of the spinal ganglion, thoracal

Invasive pain treatment	5031	503103	percutane thermolaesie van spinaal ganglion, lumbaal	percutaneous thermolysis of the spinal ganglion, lumbal
Invasive pain treatment	5031	503104	percutane thermolaesie van spinaal ganglion, sacraal	percutaneous thermolysis of the spinal ganglion, sacral
Invasive pain treatment	5031	50311	doorsnijden van zenuwwortel	dissection of the nerve root
Invasive pain treatment	5031	503110	doorsnijden van zenuwwortel, nno	dissection of the nerve root, not specified
Invasive pain treatment	5031	503111	doorsnijden van zenuwwortel, cervicaal	dissection of the nerve root, cervical
Invasive pain treatment	5031	503112	doorsnijden van zenuwwortel, thoracaal	dissection of the nerve root, thoracal
Invasive pain treatment	5031	503113	doorsnijden van zenuwwortel, lumbaal	dissection of the nerve root, lumbal
Invasive pain treatment	5031	50318	overige gespecificeerde incisie, doorsnijden of destructie van intraspinale zenuwwortel of spinaal ganglion	other specified incision, dissection or destruction of the intraspinal nerve root or spinal ganglion
Invasive pain treatment	5031	50319	incisie, doorsnijden of destructie van intraspinale zenuwwortel of spinaal ganglion, niet gespecificeerd	incision, dissection or destruction of the intraspinal nerve root or spinal ganglion, non-specific
Invasive pain treatment	5035	5035	Losmaken van adhesies van ruggenmerg en zenuwwortels	Disconnection of the adhesions of the spinal cord and nerve roots
Invasive pain treatment	5035	50350	adhesiolyse van ruggenmerg en zenuwwortels m.b.v. epidurale catheter	adhesiolysis of the spinal cord and nerve roots using epidural catheter
Invasive pain treatment	5035	50358	overig gespecificeerd losmaken van adhesies van ruggenmerg en zenuwwortels	other specified disconnection of the adhesions of the spinal cord and nerve roots
Invasive pain treatment	5035	50359	losmaken van adhesies van ruggenmerg en zenuwwortels, niet gespecificeerd	Disconnection of the adhesions of the spinal cord and nerve roots, non-specific
Invasive pain treatment	5037	5037	Injectie van destructieve stof binnen wervelkolom	Injection of destructive substance in vertebral column
Invasive pain treatment	5037	50370	No description	No description
Invasive pain treatment	5037	50371	injectie van destructieve stof, epiduraal	Injection of destructive substance, epidural
Invasive pain treatment	5037	503710	injectie van destructieve stof, epiduraal, nno	Injection of destructive substance, epidural, not specified
Invasive pain treatment	5037	503711	injectie van destructieve stof, epiduraal, cervicaal	Injection of destructive substance, epidural, cervical
Invasive pain treatment	5037	503712	injectie van destructieve stof, epiduraal, thoracaal	Injection of destructive substance, epidural, thoracal
Invasive pain treatment	5037	503713	injectie van destructieve stof, epiduraal, lumbaal	Injection of destructive substance, epidural, lumbal
Invasive pain treatment	5037	503714	injectie van destructieve stof, epiduraal, sacraal	Injection of destructive substance, epidural, sacral

Invasive pain treatment	5037	50372	injectie van destructieve stof, subarachnoïdaal	Injection of destructive substance, subarachnoidal
Invasive pain treatment	5037	50378	overige gespecificeerde injectie van destructieve stof binnen wervelkolom	other specified injection of destructive substance in vertebral column
Invasive pain treatment	5037	50379	injectie van destructieve stof binnen wervelkolom, niet gespecificeerd	Injection of the destructive substance in vertebral column, non-specific
Invasive pain treatment	5038	5038	Aanleggen, revisie en opheffen epidurale en subarachnoïdale blokkade met catheter	Construction, revision and remove epidural and subarachnoidal blockage with catheter
Invasive pain treatment	5038	50380	aanleggen van epidurale blokkade met niet gespecificeerde catheter	Construction of the epidural blockage with non-specified catheter
Invasive pain treatment	5038	503800	aanleggen van epidurale blokkade met niet gespecificeerde catheter, lokalisatie nno	Construction of the epidural blockage with non-specified catheter, location not specified
Invasive pain treatment	5038	503801	aanleggen van epidurale blokkade met niet gespecificeerde catheter, cervicaal	Construction of the epidural blockage with non-specified catheter, cervical
Invasive pain treatment	5038	503802	aanleggen van epidurale blokkade met niet gespecificeerde catheter, thoracaal	Construction of the epidural blockage with non-specified catheter, thoracal
Invasive pain treatment	5038	503803	aanleggen van epidurale blokkade met niet gespecificeerde catheter, lumbaal	Construction of the epidural blockage with non-specified catheter, lumbal
Invasive pain treatment	5038	503804	aanleggen van epidurale blokkade met niet gespecificeerde catheter, sacraal	Construction of the epidural blockage with non-specified catheter, sacral
Invasive pain treatment	5038	50381	aanleggen van epidurale blokkade met ongetunnelde catheter	Construction of the epidural blockage with untunnelled catheter
Invasive pain treatment	5038	503810	aanleggen van epidurale blokkade met ongetunnelde catheter, lokalisatie nno	Construction of the epidural blockage with untunnelled catheter, non-specified location
Invasive pain treatment	5038	503811	aanleggen van epidurale blokkade met ongetunnelde catheter, cervicaal	Construction of the epidural blockage with untunnelled catheter, cervical
Invasive pain treatment	5038	503812	aanleggen van epidurale blokkade met ongetunnelde catheter, thoracaal	Construction of the epidural blockage with untunnelled catheter, thoracal
Invasive pain treatment	5038	503813	aanleggen van epidurale blokkade met ongetunnelde catheter, lumbaal	Construction of the epidural blockage with untunnelled catheter, lumbal
Invasive pain treatment	5038	503814	aanleggen van epidurale blokkade met ongetunnelde catheter, sacraal	Construction of the epidural blockage with untunnelled catheter, sacral
Invasive pain treatment	5038	50382	aanleggen van epidurale blokkade met getunnelde catheter	Construction of the epidural blockage with tunnelled catheter
Invasive pain treatment	5038	503820	aanleggen van epidurale blokkade met getunnelde catheter, lokalisatie nno	Construction of the epidural blockage with tunnelled catheter, non-specified location

Invasive pain treatment	5038	503821	aanleggen van epidurale blokkade met getunnelde catheter zonder poortsysteem, cervicaal	Construction of the epidural blockage with tunnelled catheter without gate system, cervical
Invasive pain treatment	5038	503822	aanleggen van epidurale blokkade met getunnelde catheter met poortsys-teem, cervicaal	Construction of the epidural blockage with tunnelled catheter with gate system, cervical
Invasive pain treatment	5038	503823	aanleggen van epidurale blokkade met getunnelde catheter zonder poorts-ysteem, thoracaal	Construction of the epidural blockage with tunnelled catheter without gate system, thoracal
Invasive pain treatment	5038	503824	aanleggen van epidurale blokkade met getunnelde catheter met poortsys-teem, thoracaal	Construction of the epidural blockage with tunnelled catheter with gate system, thoracal
Invasive pain treatment	5038	503825	aanleggen van epidurale blokkade met getunnelde catheter zonder poorts-ysteem, lumbaal	Construction of the epidural blockage with tunnelled catheter without gate system, lumbal
Invasive pain treatment	5038	503826	aanleggen van epidurale blokkade met getunnelde catheter met poortsys-teem, lumbaal	Construction of the epidural blockage with tunnelled catheter with gate system, lumbal
Invasive pain treatment	5038	503827	aanleggen van epidurale blokkade met getunnelde catheter zonder poorts-ysteem, sacraal	Construction of the epidural blockage with tunnelled catheter without gate system, sacral
Invasive pain treatment	5038	503828	aanleggen van epidurale blokkade met getunnelde catheter met poortsys-teem, sacraal	Construction of the epidural blockage with tunnelled catheter with gate system, sacral
Invasive pain treatment	5038	50383	aanleggen van subarachnoïdale blokkade, lumbaal	Construction of the subarachnoidal blockage, lumbal
Invasive pain treatment	5038	503830	aanleggen van subarachnoïdale blokkade, lumbaal, nno	Construction of the subarachnoidal blockage, lumbal, not specified
Invasive pain treatment	5038	503831	aanleggen van subarachnoïdale blokkade, lumbaal, met ongetunnelde catheter	Construction of the subarachnoidal blockage, lumbal, with untunnelled catheter
Invasive pain treatment	5038	503832	aanleggen van subarachnoïdale blokkade, lumbaal, met getunnelde catheter	Construction of the subarachnoidal blockage, lumbal, with tunnelled catheter
Invasive pain treatment	5038	503833	aanleggen van subarachnoïdale blokkade, lumbaal, met getunnelde catheter met poortsysteem	Construction of the subarachnoidal blockage, lumbal, with tunnelled catheter with gate system

Invasive pain treatment	5038	50384	vervangen van epiduraal- en subarachnoïdaal catheter t.b.v. blokkade	Replacement of the epidural- and subarachnoidal catheter for the purpose of blockade
Invasive pain treatment	5038	50385	overige revisie van epiduraal- en subarachnoïdaal catheter t.b.v. blokkade	Other revision of the epidural- and subarachnoidal catheter for the purpose of blockade
Invasive pain treatment	5038	50386	verwijderen van epiduraal- en subarachnoïdaal catheter t.b.v. blokkade	Removing epidural- and subarachnoïdaal catheter for the purpose of blockage
Invasive pain treatment	5039	5039	Overige operaties van ruggenmerg en ruggenmergstructuren	Other surgeries of the spinal cord and spinal cord structures
Invasive pain treatment	5039	50390	inbrengen, revisie of verwijderen van ruggenmergstimulator	Insertion, revision or removing of the spinal cord stimulator
Invasive pain treatment	5039	503900	inbrengen van ruggenmergstimulator, nno	Insertion of the spinal cord stimulator, not specified
Invasive pain treatment	5039	503901	inbrengen van ruggenmergstimulator, cervicaal	Insertion of the spinal cord stimulator, cervical
Invasive pain treatment	5039	503902	inbrengen van ruggenmergstimulator, thoracaal	Insertion of the spinal cord stimulator, thoracal
Invasive pain treatment	5039	503903	inbrengen van ruggenmergstimulator, lumbaal	Insertion of the spinal cord stimulator, lumbal
Invasive pain treatment	5039	503904	vervangen van ruggenmergstimulator, nno	Replacement of the spinal cord stimulator, not specified
Invasive pain treatment	5039	503905	vervangen van ruggenmergstimulator, cervicaal	Replacement of the spinal cord stimulator, cervical
Invasive pain treatment	5039	503906	vervangen van ruggenmergstimulator, thoracaal	Replacement of the spinal cord stimulator, thoracal
Invasive pain treatment	5039	503907	vervangen van ruggenmergstimulator, lumbaal	Replacement of the spinal cord stimulator, lumbal
Invasive pain treatment	5039	503908	overige revisie van ruggenmergstimulator	Other revision of the spinal cord stimulator
Invasive pain treatment	5039	503909	verwijderen van ruggenmergstimulator	Removing spinal cord stimulator
Invasive pain treatment	5039	50391	revisie of verwijderen van shunt van theca spinalis	Revision or removing the shunt of the theca spinalis
Invasive pain treatment	5039	50398	overige gespecificeerde operaties van ruggenmerg en ruggenmergstructuren	other specified surgeries of the spinal cord and spinal cord structures
Invasive pain treatment	5039	50399	operaties van ruggenmerg en ruggenmergstructuren, niet gespecificeerd	surgeries of the spinal cord and spinal cord structures, non-specific
Spondylodesis	5810	5810	Spondylodese	Spondylodesis
Spondylodesis	5810	58100	craniocervicale spondylodese	craniocervical spondylodesis
Spondylodesis	5810	58101	cervicale spondylodese	cervical spondylodesis
Spondylodesis	5810	581010	cervicale spondylodese, nno	cervical spondylodesis, not specified
Spondylodesis	5810	581011	cervicale spondylodese, anterior	cervical spondylodesis, anterior
Spondylodesis	5810	581012	cervicale spondylodese, posterior	cervical spondylodesis, posterior
Spondylodesis	5810	58102	thoracale spondylodese	thoracal spondylodesis
Spondylodesis	5810	581020	thoracale spondylodese, nno	thoracal spondylodesis, not specified

Spondylodesis	5810	581021	thoracale spondylodese, anterior, nno	thoracal spondylodesis, anterior, not specified
Spondylodesis	5810	581022	thoracale spondylodese, posterolateraal	thoracal spondylodesis, postero-lateral
Spondylodesis	5810	581023	thoracale spondylodese, posterior	thoracal spondylodesis, posterior
Spondylodesis	5810	581024	thoracale spondylodese, anterior, m.b.v. endoscopie	thoracal spondylodesis, anterior, using endoscopy
Spondylodesis	5810	581025	thoracale spondylodese, anterior, open	thoracal spondylodesis, anterior, open
Spondylodesis	5810	58103	lumbale spondylodese	lumbal spondylodesis
Spondylodesis	5810	581030	lumbale spondylodese, nno	lumbal spondylodesis, not specified
Spondylodesis	5810	581031	lumbale spondylodese, anterior, nno	lumbal spondylodesis, anterior, not specified
Spondylodesis	5810	581032	lumbale spondylodese, posterolateraal	lumbal spondylodesis, postero-lateral
Spondylodesis	5810	581034	lumbale spondylodese, posterior	lumbal spondylodesis, posterior
Spondylodesis	5810	581035	lumbale spondylodese, anterior, m.b.v. endoscopie	lumbal spondylodesis, anterior, using endoscopy
Spondylodesis	5810	581036	lumbale spondylodese, anterior, open	lumbal spondylodesis, anterior, open
Spondylodesis	5810	58104	respondylodese	respondylodesis
Spondylodesis	5810	58108	overige spondylodese	other spondylodesis
Spondylodesis	5810	58109	spondylodese, niet gespecificeerd	spondylodesis, non-specific
