# Supplementary material for: The slow de‐implementation of non‐evidence‐based treatments in low back pain hospital care—Trends in treatments using Dutch hospital register data from 1991 to 2018
Source: Eur J Pain. 2022 Nov 12;27(2):212–22. doi: 10.1002/ejp.2052 (PMC10099564; doi:10.1002/ejp.2052)

Supplementary file 6. Descriptive information of the assessed low back pain (LBP) treatments.

Table depicting both absolute numbers and relative number (per 100,000 inhabitants) of all selected low back pain treatment

	**Bed rest for non-specific LBP**			**Bed rest for hernia nuclei pulposi**			**Discectomy for spinal stenosi**			**Spinal fusion**			**Invasive pain treatment**		
	**n**	**%**	**N**	**n**	**%**	**N**	**n**	**%**	**N**	**n**	**%**	**N**	**n**	**%**	**N**
Total (1991-2018)	133,631		37.6	393,790		110.8	218,635		61.5	18,051		1.7	77,259		7.2
Males	56,936	43%	32.7	216,628	55%	124.3	122,610	56%	70.3	7,265	40%	1.4	27,809	36%	5.3
Females	76,695	57%	42.3	177,162	45%	97.8	96,025	44%	53.0	10,786	60%	2.0	49,450	64%	9.1
18-25	6,254	5%	15.5	17,297	4%	42.7	10,109	5%	25.0	390	2%	0.3	600	1%	0.5
25–29	6,022	5%	19.2	20,264	5%	64.6	11,929	5%	38.0	565	3%	0.6	891	1%	1.0
30–34	10,656	8%	32.6	42,248	11%	129.2	25,347	12%	77.5	1,173	6%	1.2	2,156	3%	2.2
35–39	13,800	10%	41.2	55,293	14%	165.0	32,975	15%	98.4	2,041	11%	2.0	3,955	5%	3.9
40–44	16,015	12%	46.9	58,781	15%	172.3	34,158	16%	100.1	2,595	14%	2.5	6,397	8%	6.3
45–49	15,769	12%	47.1	53,682	14%	160.2	30,629	14%	91.4	2,611	14%	2.6	8,520	11%	8.5
50–54	13,634	10%	44.3	42,233	11%	137.1	23,436	11%	76.1	2,123	12%	2.3	9,545	12%	10.3
55–59	10,972	8%	39.9	32,501	8%	118.2	17,340	8%	63.1	1,755	10%	2.1	9,554	12%	11.6
60–64	9,231	7%	38.1	24,364	6%	100.5	12,242	6%	50.5	1,399	8%	1.9	8,091	10%	11.1
65–69	8,541	6%	40.9	19,308	5%	92.4	9,106	4%	43.6	1,281	7%	2.0	7,881	10%	12.6
70–74	7,771	6%	45.8	14,264	4%	84.1	6,314	3%	37.3	1,144	6%	2.3	7,654	10%	15.1
75–79	6,858	5%	52.5	8,756	2%	67.0	3,593	2%	27.5	673	4%	1.7	6,434	8%	16.4
80–84	4,842	4%	53.5	3,694	1%	40.8	1,184	1%	13.1	257	1%	1.0	4,006	5%	14.8
85+	3,266	2%	44.6	1,105	0%	15.1	273	0%	3.7	44	0%	0.2	1,575	2%	7.2

n = Absolute number of low back pain treatments
N = Relative number of low back pain treatment (per 100,000 inhabitants)

Figure depicting the use of relative low back pain treatments (per 100,000 inhabitants), stratified by sex (upper panel) and age category (lower panel).

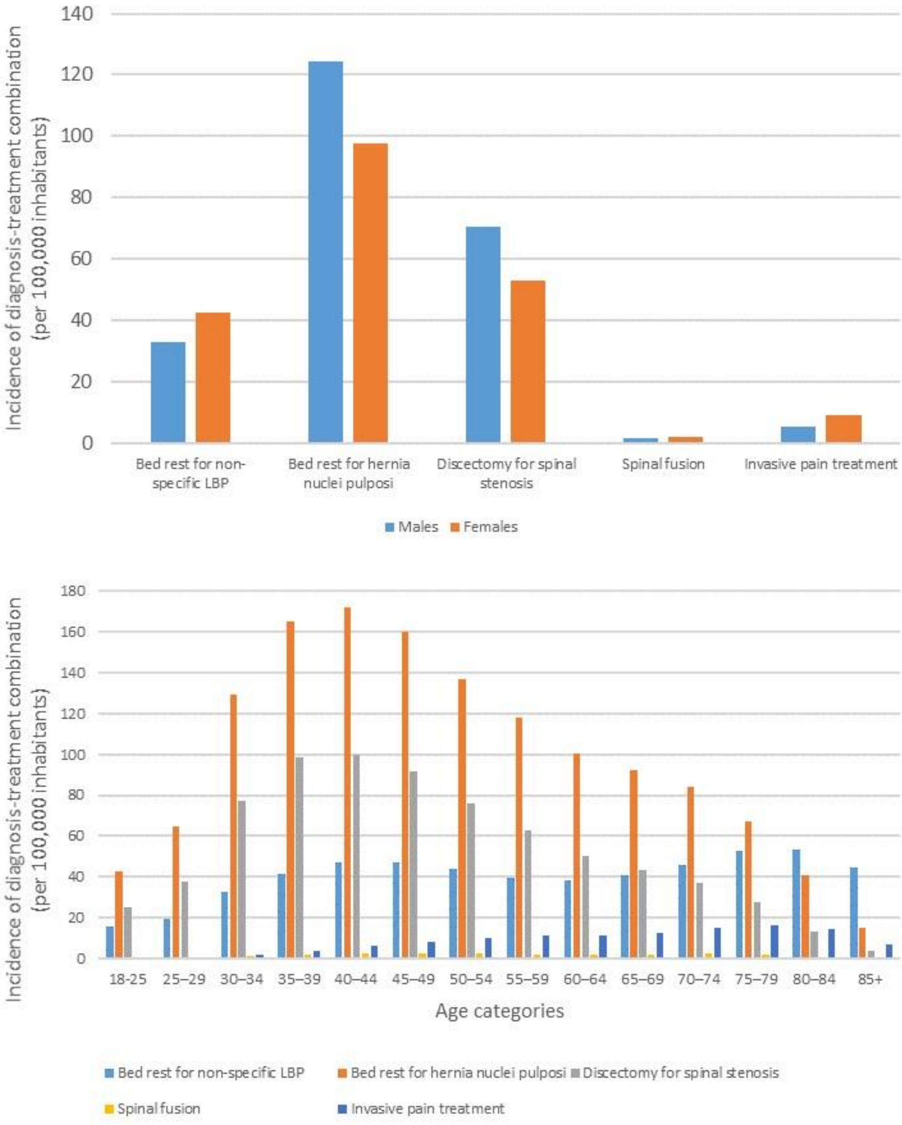

Supplement: Supplementary file 6 — Supplementary file S6 [file EJP-27-212-s005.pdf]
