# Supplementary material for: The slow de‐implementation of non‐evidence‐based treatments in low back pain hospital care—Trends in treatments using Dutch hospital register data from 1991 to 2018
Source: Eur J Pain. 2022 Nov 12;27(2):212–22. doi: 10.1002/ejp.2052 (PMC10099564; doi:10.1002/ejp.2052)
Supplement: Supplementary file 7 — Supplementary file S7 [file EJP-27-212-s003.pdf]

Supplementary file 7. Percentage (%) of de-implementation during the study period and, if at all, time until successful de-implementation (using a 84% threshold). De-implementation with the first year of study (1991) as reference and with the first landmark publication (see Supplementary file 2) as reference are reported.

**Treatments**	**De-implementation with 1991 as reference**		**De-implementation with first landmark as reference**		
	**% de-implementation**	**Time until successful de-implementation**	**First landmark**	**% de-implementation**	**Time until successful de-implementation**
Bed rest for non-specific LBP	94%	23 year	1996	91%	18 year
Bed rest for hernia nuclei pulposi	83%	Not	1995	81%	Not
Discectomy for spinal stenosis	89%	25 years	2000	86%	17 years
Spinal fusion	78%	Not	2000	85%	19 years
Invasive pain treatment	35%	Not	1999	75%	Not
