# Supplementary material for: The slow de‐implementation of non‐evidence‐based treatments in low back pain hospital care—Trends in treatments using Dutch hospital register data from 1991 to 2018
Source: Eur J Pain. 2022 Nov 12;27(2):212–22. doi: 10.1002/ejp.2052 (PMC10099564; doi:10.1002/ejp.2052)

Supplementary file 8. Sensitivity analyses.

First table shows trends in absolute treatments, from Poisson (upper panel) and GEE (lower panel) regression analysis. Moreover, analyses for spinal fusion and invasive pain treatment were stratified by three diagnosis categories: non-specific low back pain (LBP), degenerative low back problems and lumbar spinal stenosis.

	**Poisson GLM with robust standard errors**				
	**Overall**	**Period II vs I[1]**		**Period III vs II[1]**	
	**p-value**	**Adjusted IRR [95% CI]**	**p-value**	**Adjusted IRR [95% CI]**	**p-value**
Bed rest for non-specific LBP	<0.001	0.47 [0.44 0.51]	<0.001	0.46 [0.42 0.50]	<0.001
Bed rest for hernia nuclei pulposi	<0.001	0.72 [0.68 0.76]	<0.001	0.52 [0.49 0.55]	<0.001
Discectomy for lumbar spinal stenosis	<0.001	0.61 [0.58 0.64]	<0.001	0.28 [0.24 0.32]	<0.001
Lumbar fusion/laminectomy					
Total	<0.001	1.13 [1.04 1.22]	0.005	0.45 [0.40 0.51]	<0.001
Non-specific LBP	<0.001	0.54 [0.48 0.62]	<0.001	0.22 [0.17 0.28]	<0.001
Degenerative low back problems	<0.001	**1.05 [0.97 1.13]**	**0.52**	0.25 [0.21 0.31]	<0.001
Lumbar spinal stenosis	<0.001	2.17 [1.95 2.41]	<0.001	0.89 [0.80 0.99]	0.050
Invasive pain treatment					
Total	<0.001	2.21 [2.06 2.37]	<0.001	0.66 [0.59 0.74]	<0.001
Non-specific LBP	<0.001	2.37 [2.19 1.63]	<0.001	0.60 [0.53 0.68]	<0.001
Degenerative low back problems	<0.001	0.61 [0.53 0.70]	<0.001	1.54 [1.33 1.78]	<0.001
Lumbar spinal stenosis	<0.001	2.32 [1.82 2.95]	<0.001	2.78 [2.41 3.21]	<0.001
	**GEE[2]**				
	**Overall**	**Period II vs I[1]**		**Period III vs II[1]**	
	**p-value**	**Adjusted IRR [95% CI]**	**p-value**	**Adjusted IRR [95% CI]**	**p-value**
Bed rest for non-specific LBP	<0.001	0.47 [0.43 0.52]	<0.001	0.46 [0.40 0.52]	<0.001
Bed rest for hernia nuclei pulposi	<0.001	0.72 [0.69 0.75]	<0.001	0.52 [0.47 0.57]	<0.001
Discectomy for lumbar spinal stenosis	<0.001	0.61 [0.56 0.67]	<0.001	0.27 [0.28 0.31]	<0.001
Lumbar fusion/laminectomy					
Total	<0.001	**1.13 [0.95 1.34]**	**0.36**	0.45 [0.37 0.57]	<0.001
Non-specific LBP	<0.001	0.54 [0.47 0.63]	<0.001	0.22 [0.17 0.28]	<0.001
Degenerative low back problems	<0.001	**1.05 [0.91 1.20]**	**1.00**	0.25 [0.22 0.29]	<0.001
Lumbar spinal stenosis	<0.001	2.17 [1.92 2.46]	<0.001	0.89 [0.82 0.97]	0.022
Invasive pain treatment					
Total	<0.001	2.21 [1.97 2.47]	<0.001	0.66 [0.61 0.71]	<0.001
Non-specific LBP	<0.001	2.37 [2.12 2.64]	<0.001	0.60 [0.58 0.65]	<0.001
Degenerative low back problems	<0.001	0.61 [0.51 0.74]	<0.001	1.64 [1.36 1.97]	<0.001

Lumbar spinal stenosis	<0.001	2.39 [1.67 3.21]	<0.001	2.78 [2.35 3.30]	<0.001

[1] Generalized estimation equation (GEE) with independent working correlation structure and robust standard errors to account for possible correlation of outcomes within same age group and sex combination in different years.

[2] Cells contain incidence rate ratios (IRR), their 95% confidence intervals and p-values for comparing the incidence rates in the two periods. P-values are Bonferroni corrected p-values (corrected for two pairwise comparisons).

Second table shows trends in relative treatments, from Poisson (upper panel) and GEE (lower panel) regression analysis with the logarithm of population size used as offset. Moreover, analyses for spinal fusion and invasive pain treatment were stratified by three diagnosis categories: non-specific low back pain (LBP), degenerative low back problems and lumbar spinal stenosis.

	**Poisson GLM with logarithm of population size used as offset and robust standard errors**				
	**Overall**	**Period II vs I[1]**		**Period III vs II[1]**	
	**p-value**	**Adjusted IRR [95% CI]**	**p-value**	**Adjusted IRR [95% CI]**	**p-value**
Bed rest for non-specific LBP	< 0.001	0.44 [0.41 0.48]	<0.001	0.42 [0.38 0.45]	<0.001
Bed rest for hernia nuclei pulposi	< 0.001	0.68 [0.65 0.71]	<0.001	0.48 [0.46 0.51]	<0.001
Discectomy for lumbar spinal stenosis	< 0.001	0.57 [0.54 0.59]	<0.001	0.27 [0.23 0.31]	<0.001
Lumbar fusion/laminectomy					
Total	<0.001	1.02 [0.95 1.09]	1.0	0.43 [0.38 0.48]	<0.001
Non-specific LBP	<0.001	0.50 [0.44 0.56]	<0.001	0.21 [0.17 0.27]	<0.001
Degenerative low back problems	<0.001	0.96 [0.90 1.03]	0.51	0.25 [0.21 0.30]	<0.001
Lumbar spinal stenosis	<0.001	1.90 [1.72 2.10]	<0.001	0.77 [0.70 0.84]	<0.001
Invasive pain treatment					
Total	<0.001	1.94 [1.83 2.07]	<0.001	0.58 [0.52 0.66]	<0.001
Non-specific LBP	<0.001	2.09 [1.95 2.23]	<0.001	0.53 [0.47 0.60]	<0.001
Degenerative low back problems	<0.001	0.54 [0.47 0.62]	<0.001	1.37 [1.18 1.59]	<0.001
Lumbar spinal stenosis	<0.001	2.03 [1.60 2.56]	<0.001	2.31 [2.00 2.66]	< 0.001
	**GEE with logarithm of population size used as offset[2]**				
	**Overall**	**Period II vs I[1]**		**Period III vs II[1]**	
	**p-value**	**Adjusted IRR [95% CI]**	**p-value**	**Adjusted IRR [95% CI]**	**p-value**
Bed rest for non-specific LBP	<0.001	0.44 [0.41 0.48]	<0.001	0.42 [0.38 0.46]	<0.001
Bed rest for hernia nuclei pulposi	<0.001	0.68 [0.66 0.70]	<0.001	0.48 [0.46 0.51]	<0.001
Discectomy for lumbar spinal stenosis	<0.001	0.57 [0.54 0.59]	<0.001	0.27 [0.25 0.29]	<0.001
Lumbar fusion/laminectomy					
Total	<0.001	1.02 [0.88 1.19]	1.0	0.43 [0.36 0.51]	<0.001
Non-specific LBP	<0.001	0.50 [0.44 0.56]	<0.001	0.21 [0.17 0.26]	<0.001
Degenerative low back problems	<0.001	0.96 [0.86 1.07]	0.93	0.25 [0.23 0.27]	<0.001
Lumbar spinal stenosis	<0.001	1.90 [1.70 2.13]	<0.001	0.77 [0.72 0.81]	<0.001
Invasive pain treatment					
Total	<0.001	1.94 [1.76 2.14]	<0.001	0.58 [0.54 0.62]	<0.001
Non-specific LBP	<0.001	2.08 [1.90 2.29]	<0.001	0.53 [0.50 0.57]	<0.001
Degenerative low back problems	<0.001	0.54 [0.46 0.64]	<0.001	1.37 [1.24 1.52]	<0.001
Lumbar spinal stenosis	<0.001	2.03 [1.46 2.81]	<0.001	2.31 [1.98 2.67]	<0.001

[1] Generalized estimation equation (GEE) with independent working correlation structure and robust standard errors to account for possible correlation of outcomes within same age group and sex combination in different years.
[2] Cells contain incidence rate ratios (IRR), their 95% confidence intervals and p-values for comparing the incidences in the two periods. P-values are Bonferroni corrected p-values (corrected for two pairwise comparisons).

Figure showing the yearly absolute use of spinal fusion and invasive pain treatment stratified for diagnoses: degenerative low back pain, spinal stenosis and non-specific low back pain.

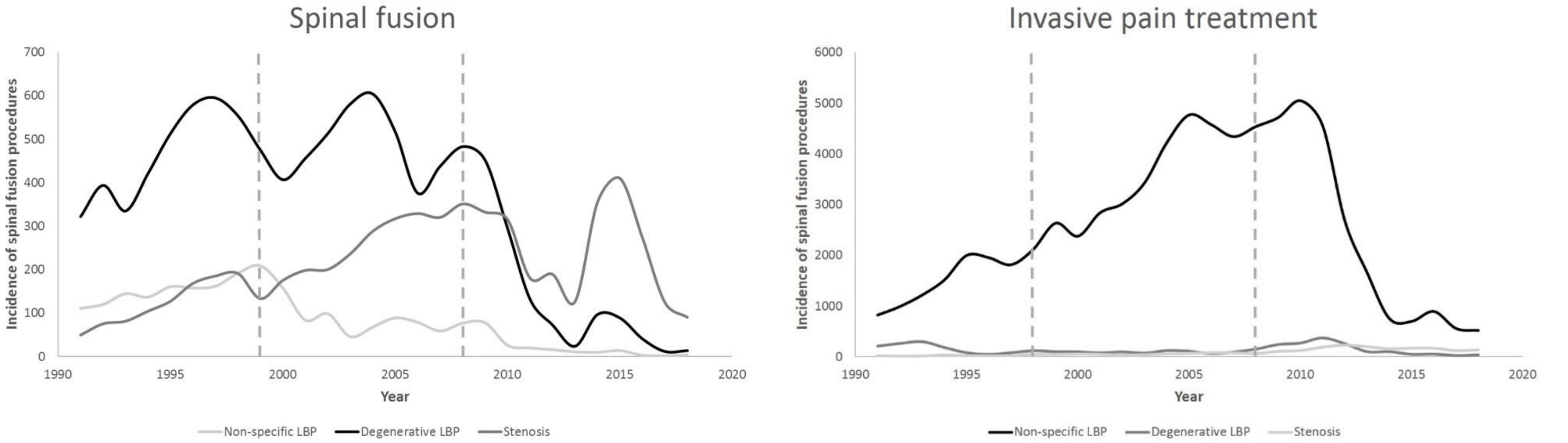

Figure showing the yearly relative use (per 100,000 inhabitants) of all selected low back pain treatments. Vertical dashed lines depict the boundaries for the three time-periods that are used for analyses.

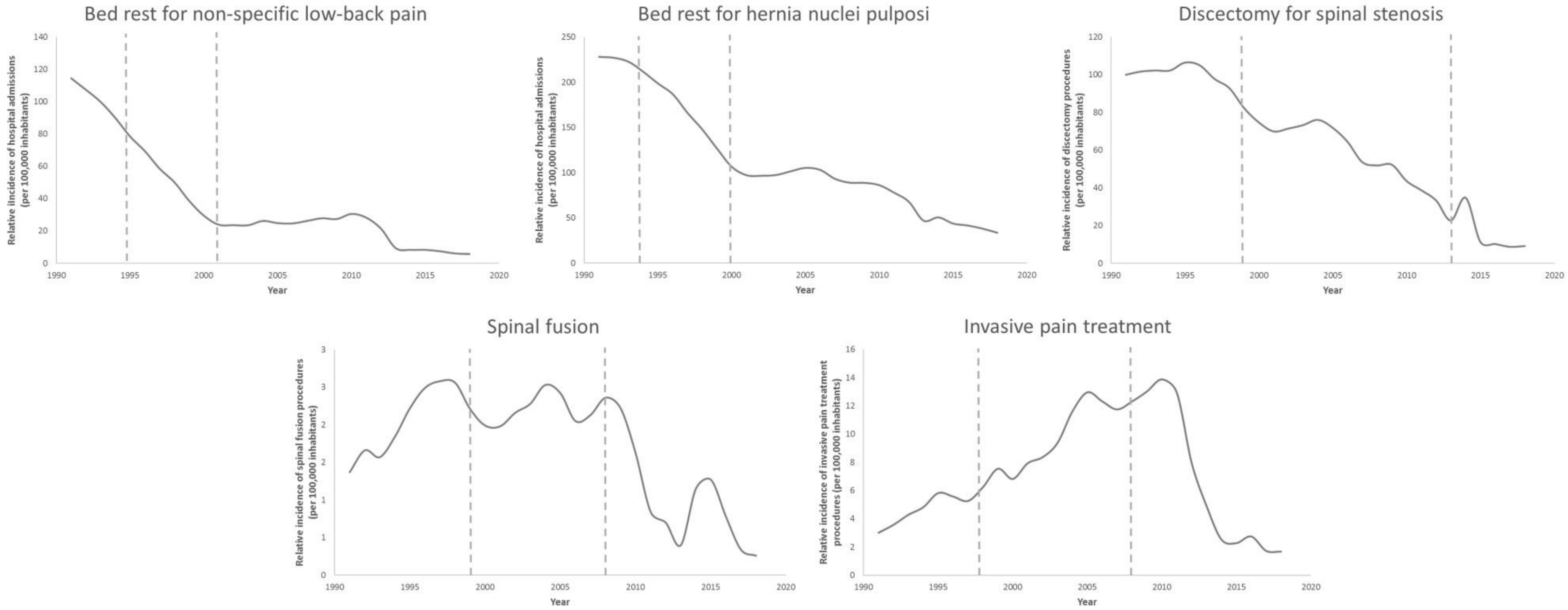

Supplement: Supplementary file 8 — Supplementary file S8 [file EJP-27-212-s006.pdf]
